# Mapping the research landscape of antibody therapy for Ebola virus disease: a bibliometric analysis

**DOI:** 10.3389/fimmu.2026.1919677

**Published:** 2026-09-03

**Authors:** Zengwei Kou, Yunsheng Liu, Wujie Qian, Sihan Zhang, Shulei Lou, Da Shao

**Affiliations:** 1 Research Center of Translational Medicine, Shanghai Children’s Hospital, School of Medicine, Shanghai Jiao Tong University, Shanghai, China; 2 College of Basic Medicine and Forensic Medicine, Henan University of Science and Technology, Luoyang, China; 3 Cancer Center, Shenzhen Hospital (Futian) of Guangzhou University of Chinese Medicine, Shenzhen, China; 4 Central Laboratory, Linyi People’s Hospital, Linyi, China

**Keywords:** antibody therapy, bibliometric analysis, Ebola virus disease, Ebola virus, immunotherapy, monoclonal antibody

## Abstract

**Background:**

Antibody-based therapies have become a cornerstone of Ebola virus disease (EVD) treatment and prevention, particularly following the successful clinical development of monoclonal antibody therapeutics against Zaire ebolavirus. However, despite substantial advances, the global research landscape, collaboration patterns, and evolving thematic trends of Ebola antibody therapy have not been systematically characterized. The ongoing 2026 Bundibugyo ebolavirus outbreak, for which no licensed vaccines or therapeutics currently exist, further underscores the importance of understanding the development trajectory of this field.

**Methods:**

Publications related to antibody therapy for EVD published between 1990 and 2026 were retrieved from the Web of Science Core Collection, PubMed, and Scopus databases. Following data integration, format conversion, and deduplication, a total of 1,350 unique publications were included. Bibliometric analyses were conducted using CiteSpace, VOSviewer, and Bibliometrix, complemented by Google Trends data to evaluate public interest and outbreak-related attention.

**Results:**

The field exhibited dynamic outbreak-driven growth, with major publication surges corresponding to the 2014–2016 West African Ebola epidemic and the period surrounding 2020. Although annual publication output declined after 2020, renewed public attention emerged during the 2026 Bundibugyo outbreak. The United States dominated both publication productivity and citation impact, followed by China, the United Kingdom, and Canada. Harvard University, the University of Texas System, and Scripps Research Institute were identified as leading institutions. Keyword co-occurrence and clustering analyses revealed five major thematic domains: fundamental virology and glycoprotein research, immunity and vaccination, infection and therapeutic development, cross-viral research involving COVID-19 and HIV, and clinically oriented studies focused on neutralizing antibodies, clinical trials, and convalescent plasma. Temporal analyses demonstrated a clear evolution from basic virology toward translational and clinical applications.

**Conclusions:**

Ebola antibody therapy research has undergone substantial expansion over the past three decades, driven largely by major outbreak events and advances in antibody engineering technologies. The current Bundibugyo outbreak highlights persistent vulnerabilities in existing countermeasure strategies and emphasizes the urgent need for broadly protective pan-ebolavirus antibody therapeutics. These findings provide a comprehensive overview of the intellectual structure, global collaborations, research hotspots, and future directions of antibody-based interventions against Ebola virus disease.

## 1 Introduction

As of June 2026, the Democratic Republic of the Congo (DRC) and Uganda are grappling with a rapidly escalating Ebola outbreak caused by the Bundibugyo virus, a rare species of Ebola virus for which no licensed vaccines or specific therapeutics currently exist (1, 2). First declared a public health emergency of international concern on May 20, 2026, the outbreak has since expanded across three provinces in the DRC-Ituri, North Kivu, and South Kivu-with confirmed cases also reported in Uganda (3). As of June 2, 2026, the DRC and Uganda recorded over 378 confirmed cases and 63 deaths, with a case fatality rate of approximately 16% (4). The outbreak is occurring in a challenging context marked by ongoing conflict, mass displacement, strained healthcare infrastructure, and significant population movement, all of which have complicated containment efforts. The World Health Organization has warned that the true scale of the epidemic may be far larger than official figures suggest, citing “blind spots” in high-risk areas and contact tracing coverage that remains well below the agency’s target (WHO). The urgency of this situation underscores the critical need for effective therapeutic interventions against Ebola virus disease, particularly for strains such as Bundibugyo that lack approved medical countermeasures.

Ebola virus disease, first identified in 1976 near the Ebola River in what is now the Democratic Republic of Congo, is a severe, often fatal illness caused by viruses of the genus *Orthoebolaviru*s within the family Filoviridae (5, 6). Six species have been identified, of which four-Zaire ebolavirus, Sudan ebolavirus, Bundibugyo ebolavirus, and Taï Forest ebolavirus-are known to cause disease in humans (7, 8). The virus is transmitted to humans through contact with infected wildlife, such as fruit bats and nonhuman primates, and spreads among humans via direct contact with bodily fluids of infected individuals, contaminated surfaces, or infected healthcare settings. Case fatality rates have varied widely across outbreaks, ranging from 34% to as high as 79% depending on the viral species and the quality of medical care available (9). Over the past five decades, Ebola has caused numerous outbreaks in Central and West Africa, with the 2014–2016 epidemic in West Africa-primarily driven by the Zaire species-representing the largest and most devastating in history, resulting in over 28,000 cases and more than 11,000 deaths (10–12). The discovery of Ebola virus was followed by decades of basic research focused on understanding its virology, pathogenesis, and transmission dynamics, culminating in the development of two licensed vaccines (rVSV-ZEBOV and Ad26.ZEBOV/MVA-BN-Filo) and several experimental therapeutics, including monoclonal antibody cocktails such as ZMapp, REGN-EB3, and mAb114, which have demonstrated efficacy primarily against the Zaire species (13, 14). However, as the current outbreak starkly illustrates, the Bundibugyo species-for which no approved vaccines or therapeutics exist-remains a formidable challenge.

Antibody-based therapies have emerged as one of the most promising approaches for the treatment of Ebola virus disease (15–17). Monoclonal antibodies (mAbs) offer several advantages over conventional antiviral drugs, including high target specificity, favorable safety profiles, and the potential for rapid development and deployment in outbreak settings. The success of antibody cocktails such as REGN-EB3 and mAb114 in clinical trials during the 2018–2020 Ebola outbreak in the DRC, where they significantly reduced mortality compared to ZMapp, has galvanized interest in antibody-based interventions (18, 19). These therapeutics function through multiple mechanisms, including direct neutralization of the virus by blocking receptor binding or membrane fusion, antibody-dependent cellular cytotoxicity, and complement-mediated lysis. Beyond the Zaire species, researchers have also explored broadly neutralizing antibodies targeting conserved epitopes across multiple ebolavirus species, as well as engineered antibody formats such as bispecific antibodies, nanobodies, and single-domain antibodies that may offer enhanced potency, breadth, or manufacturability (20–24). Despite these advances, significant gaps remain in our understanding of the antibody response to Ebola virus, the correlates of protection, and the optimal strategies for antibody-based prevention and treatment, particularly for rare species such as Bundibugyo. The current outbreak has reignited urgency for developing antibody therapies that are effective across ebolavirus species and can be rapidly deployed in resource-limited settings.

Bibliometric analysis has become an indispensable tool for systematically mapping the intellectual structure, historical evolution, and emerging frontiers of scientific research fields (25–28). By quantitatively analyzing large corpora of scholarly publications-including trends in annual output, collaboration networks among countries and institutions, citation patterns, and keyword co-occurrence-bibliometric methods can reveal the underlying dynamics of a research domain that are often obscured in traditional narrative reviews. These approaches enable researchers to identify core journals, influential authors, landmark publications, and emerging topics, while also uncovering gaps and opportunities for future investigation. In the context of infectious disease research, bibliometric analyses have been increasingly employed to inform research prioritization and public health policy. For Ebola virus research specifically, several bibliometric studies have examined publication trends, collaboration patterns, and research hotspots, yet none has focused specifically on antibody therapy-a domain that has witnessed remarkable growth and transformation over the past decade, particularly in response to successive outbreaks and the urgent need for effective medical countermeasures.

To address this gap, we conducted a comprehensive bibliometric analysis of antibody therapy research for Ebola virus disease, leveraging publications from the Web of Science Core Collection, PubMed, and Scopus spanning 1990 to 2026. This study aims to: (1) characterize the global publication and citation landscape; (2) identify the most productive and influential countries/regions, institutions, journals, and authors; (3) map the thematic structure and emerging research hotspots; (4) examine temporal trends and outbreak-driven research surges; and (5) highlight translational opportunities and knowledge gaps to inform future research and public health preparedness. By providing a systematic and data-driven synthesis, this study serves as a valuable reference for researchers, clinicians, and policymakers seeking to understand the historical trajectory, current landscape, and future directions of antibody therapy for Ebola virus disease.

## 2 Methods and materials

### 2.1 Data sources and search strategy

This study employed a multi-database retrieval approach, encompassing the Web of Science Core Collection, PubMed, and Scopus.

For the Web of Science Core Collection, the following search string was applied in the Topic (TS) field:

TS=(((Ebola* OR “Ebola virus” OR EBOV OR “Orthoebolavirus*” OR “Ebolavirus*” OR “Zaire ebolavirus” OR “Sudan ebolavirus” OR “Bundibugyo ebolavirus” OR “Tai Forest ebolavirus” OR “Reston ebolavirus” OR SUDV OR BDBV OR TAFV OR RESTV) AND (“therapeutic antibody*” OR “monoclonal antibody*” OR mAb* OR “neutralizing antibody*” OR “recombinant antibody*” OR “bispecific antibody*” OR “nanobody*” OR “single-domain antibody*” OR “immune therapy” OR “antibody therapy” OR immunotherap*)) NOT (Marburg* OR MARV OR “Lake Victoria” OR “Filoviridae”)).

For PubMed, the search strategy was formulated as follows:

((“Ebolavirus”[Mesh] OR “Ebola virus”[MeSH] OR “Zaire ebolavirus”[Mesh] OR “Sudan ebolavirus”[Mesh] OR “Bundibugyo ebolavirus”[Mesh]) OR (Ebola*[tiab] OR “Ebola virus”[tiab] OR EBOV[tiab] OR “Orthoebolavirus*”[tiab] OR “Ebolavirus*”[tiab] OR “Zaire ebolavirus”[tiab] OR “Sudan ebolavirus”[tiab] OR “Bundibugyo ebolavirus”[tiab] OR “Tai Forest ebolavirus”[tiab] OR “Reston ebolavirus”[tiab] OR SUDV[tiab] OR BDBV[tiab] OR TAFV[tiab] OR RESTV[tiab])) AND ((“Antibodies, Monoclonal”[Mesh] OR “Antibodies, Neutralizing”[Mesh] OR “Immunotherapy”[Mesh]) OR (“therapeutic antibody*”[tiab] OR “monoclonal antibody*”[tiab] OR mAb*[tiab] OR “neutralizing antibody*”[tiab] OR “recombinant antibody*”[tiab] OR “bispecific antibody*”[tiab] OR “nanobody*”[tiab] OR “single-domain antibody*”[tiab] OR “immune therapy”[tiab] OR “antibody therapy”[tiab] OR immunotherap*[tiab])) NOT (Marburgvirus[Mesh] OR Marburg*[tiab] OR MARV[tiab] OR “Lake Victoria”[tiab] OR Filoviridae[Mesh]).

For Scopus, the search was performed in the TITLE-ABS-KEY field using the following string:

TITLE-ABS-KEY((Ebola* OR “Ebola virus” OR EBOV OR “Orthoebolavirus*” OR “Ebolavirus*” OR “Zaire ebolavirus” OR “Sudan ebolavirus” OR “Bundibugyo ebolavirus” OR “Tai Forest ebolavirus” OR “Reston ebolavirus” OR SUDV OR BDBV OR TAFV OR RESTV) AND (“therapeutic antibody*” OR “monoclonal antibody*” OR mAb* OR “neutralizing antibody*” OR “recombinant antibody*” OR “bispecific antibody*” OR “nanobody*” OR “single-domain antibody*” OR “immune therapy” OR “antibody therapy” OR immunotherap*)) AND NOT (Marburg* OR MARV OR “Lake Victoria” OR Filoviridae).

All research was conducted without language, publication date, or document type restrictions to ensure maximum coverage and inclusivity.

### 2.2 Data integration, cleaning, and deduplication

Data preprocessing was done as previously described with small modification. Records retrieved from Web of Science were downloaded as plain text (.txt) files, from PubMed as.nbib files, and from Scopus as.csv files.

For PubMed and Scopus records, the original file formats were first converted to Web of Science-compatible plain text format. Notably, direct format conversion of Scopus data using CiteSpace resulted in substantial data loss. To circumvent this issue, we employed the Bibliometric-Data-Consolidation-Tool (available at: https://github.com/LeoMengTCM/Bibliometric-Data-Consolidation-Tool) for robust format conversion prior to merging.

Following our previously established workflow (27–29), data merging and deduplication were performed using a customized processing pipeline. Specifically, the converted plain-text records were first consolidated into a single file using the Linux *cat* command. This merged file was then processed using a customized R script for deduplication, which prioritized Digital Object Identifiers (DOIs) for unique identification; in cases where a DOI was missing, the script matched records based on a combination of Title, First Author, and Publication Year. Subsequently, a customized Python script was applied to filter out retracted publications, errata, and news items. Following this automated filtering, two of our authors (K.Z.W. and L.Y.S.) manually screened the remaining records to exclude irrelevant publications. After consolidation and deduplication, the integrated dataset was subjected to bibliometric analyses and visualizations using Bibliometrix, CiteSpace, and VOSviewer.

### 2.3 Google Trends analysis

Google Trends data was acquired and analyzed as previously described (26). For the search term “Ebola” were downloaded from the official Google Trends website (https://trends.google.com/trends/) as a CSV file.

For temporal trend analysis of public interest, the peak search volume within the study period was normalized to 100%, and relative search volumes at other time points were semi-quantitatively compared against this baseline for visualization. Annual relative search volumes were subsequently calculated and plotted.

For spatial visualization of regional/country-level search interest over the past five years, a custom R script was developed. Raw CSV data were imported, followed by cleaning and standardization of region names and corresponding search volume values. The processed data were then linked to a spatial polygon data frame using the rworldmap package. A geographic map was generated, with Ebola-related search volumes represented as red-scaled points (point size = value/50). A legend was added, and the base map was rendered using the mapCountryData function with a white color palette and fixed-width categorization.

## 3 Results

### 3.1 Overall publication trends and public interest

**Figure 1:**
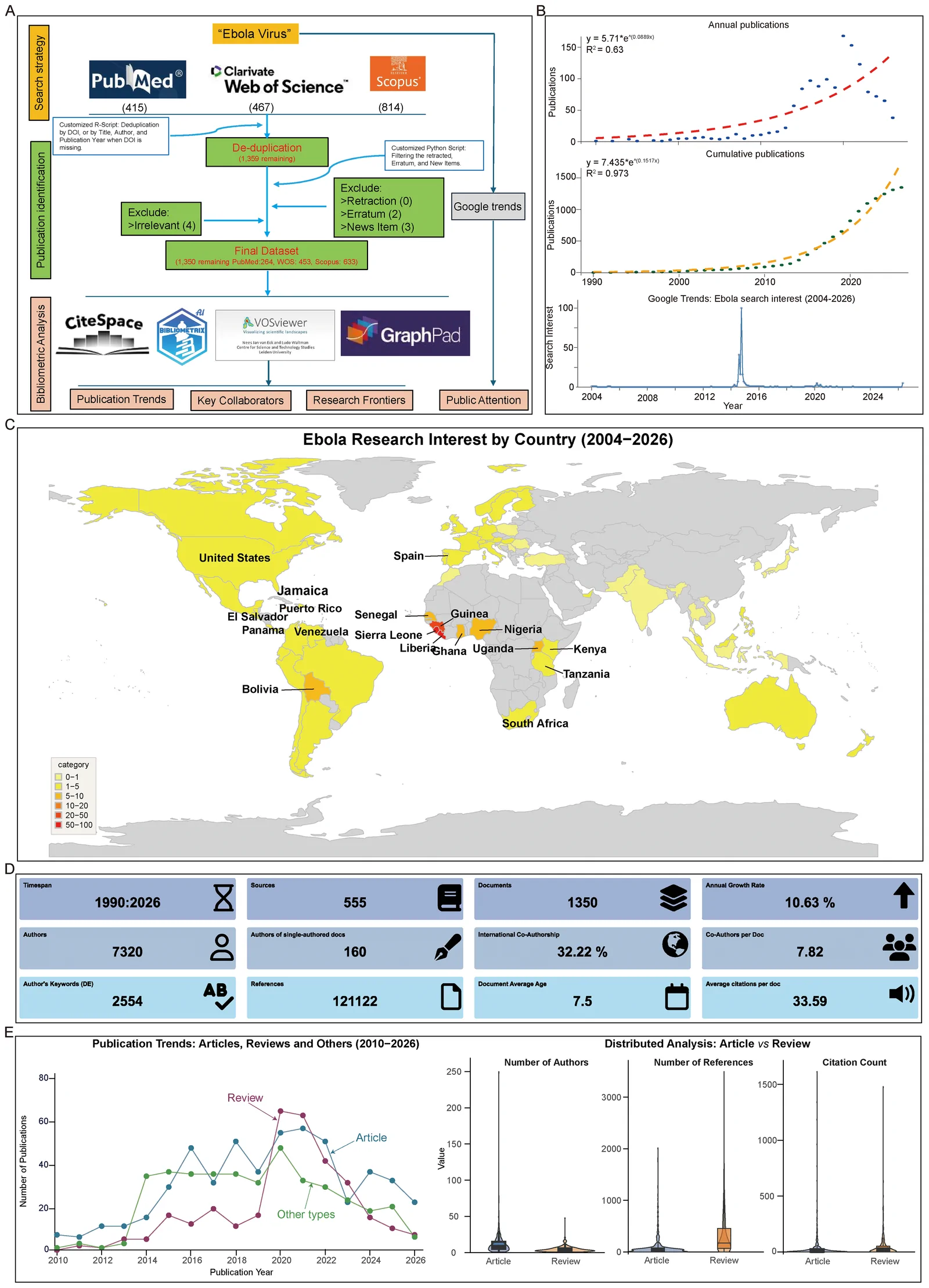
Overview of the literature search and general characteristics of the included publications. **(A)** Flowchart illustrating database search, format conversion, merging, deduplication, and screening processes across Web of Science, PubMed, and Scopus. **(B)** Trends in annual and cumulative publications on antibody therapy for Ebola virus disease from 1990 to 2026, overlaid with Google Trends search interest for “Ebola” (relative search volume normalized to 100%). **(C)** Geographic distribution of Google Trends search interest for “Ebola” during 2004–2026, with red-scaled points indicating relative search volume by country/region. **(D)** Summary statistics of the final corpus: total publications, publication time span, number of journals, authors, international collaborations, average citations per document, and author productivity. **(E)** Annual distribution of document types (original articles vs. review articles) and their respective citation trends over time.

Following the search strategy and retrieval process, we obtained 415, 467, and 814 records from PubMed, Web of Science, and Scopus, respectively. After rigorous format conversion, merging, and deduplication, a final corpus of 1,350 unique publications was established. Bibliometric analyses and visualizations were performed using CiteSpace, VOSviewer, Bibliometrix, and GraphPad Prism (**Figure 1A**).

Both annual and cumulative publication outputs exhibited an overall upward trend prior to 2020. A notable peak in annual publications occurred between 2012 and 2016, which corresponded to a contemporaneous surge in public interest as reflected by Google Trends data(**Figure 1B**). Following this period, annual output declined briefly but soon rebounded into a phase of rapid growth, reaching its historical zenith around 2020. Thereafter, annual publications exhibited a declining trajectory. Consequently, the annual publication growth did not conform to an exponential model (R² = 0.63). In contrast, cumulative publication output followed a pronounced exponential growth pattern. Prior to 2020, the cumulative curve could be well fitted by the model Y = 7.435 × e^(0.1517x), with R² = 0.973 (**Figure 1B**). However, due to the post-2020 decline in annual output, the cumulative curve has shown signs of entering a plateau phase.

Google Trends data revealed two major peaks in public search interest: the first during 2014-2016, and a second during 2020-2021. Notably, after 2026, search interest has shown signs of resurgence (**Figure 1B**), likely associated with regional or large-scale epidemic events and public health emergencies (30, 31). A geographic breakdown of Ebola-related search interest during 2023–2026 identified Sierra Leone, Liberia, Guinea, and Uganda (all near-equatorial African nations) as the most attentive regions, followed by Bolivia in South America and South Africa in southern Africa (**Figure 1C**).

Among the 1,350 retrieved publications, the time span extended from 1990 to 2026, distributed across 555 specialized journals and contributed by 7,320 authors, with an annual publication growth rate of 10.63%. The average number of authors per article was 7.82, and only 160 articles were single-authored (**Figure 1D**). In terms of document types, original articles consistently outnumbered review articles in most years, with the exceptions of 2020, 2021, and 2023. This pattern is also reflected in the overall summary statistics: the total number of articles (including journal articles, article; book chapter articles; and proceedings papers) exceeded that of reviews (including conference reviews, reviews, and review chapters) across the entire study period (**Supplementary Table 1**).

Citation analysis revealed that original articles exhibited greater variance in citation counts, whereas review articles generally received higher median citations. Both document types displayed largely congruent temporal citation trends (**Figure 1E**).

### 3.2 High-impact countries and their publication trends

**Figure 2:**
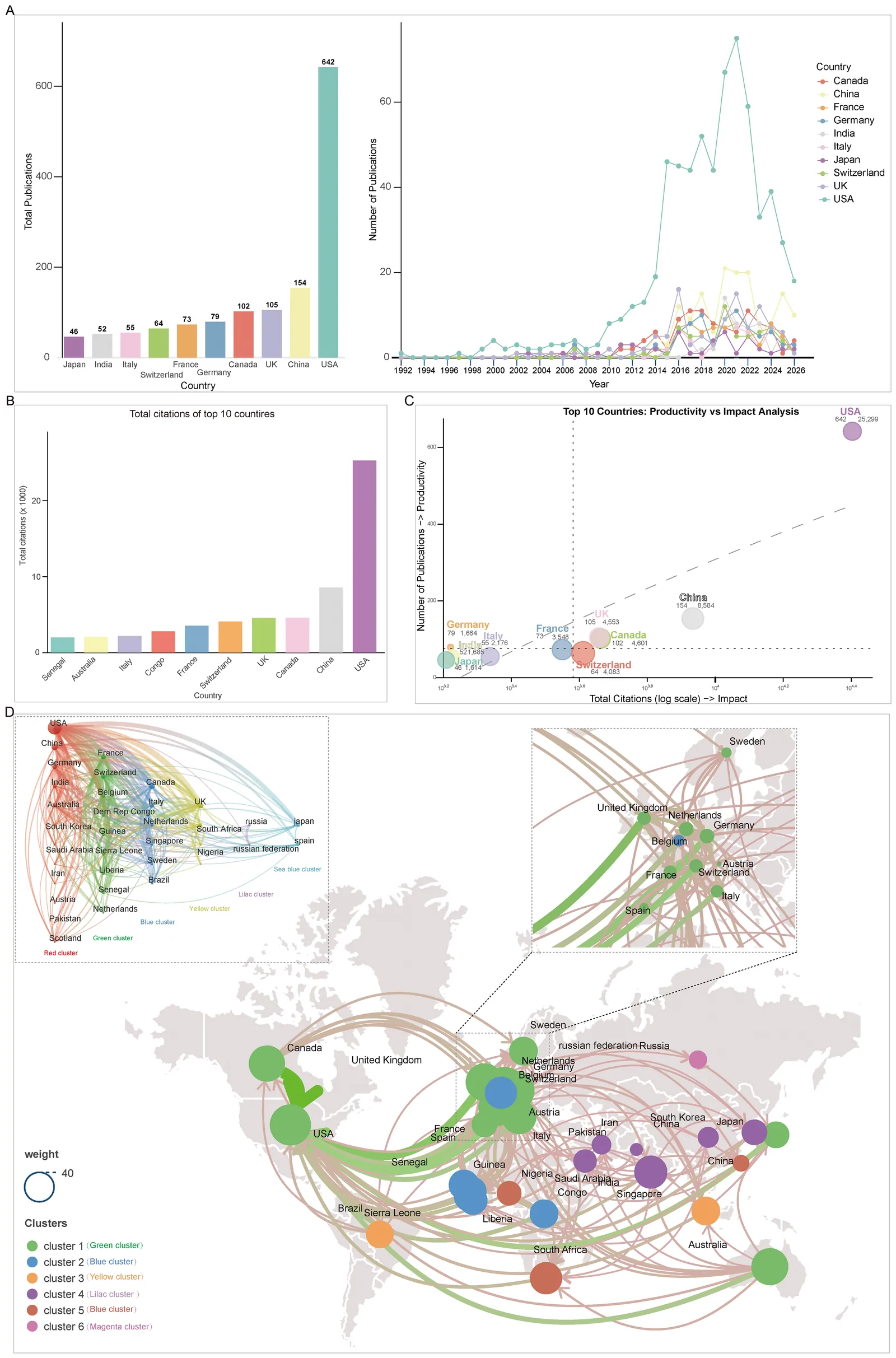
Country-level publication performance and collaboration networks. **(A)** Annual publication output of the top 10 most productive countries based on corresponding author affiliations. **(B)** Total citations of the top 10 most-cited countries, with Senegal highlighted as a notable contributor despite not ranking in the top 10 by publication volume. **(C)** Two-dimensional scatter plot of total publications versus total citations for the top 10 most productive countries. **(D)** International collaboration network among countries, with node size representing publication volume and edge thickness indicating collaboration strength. Clusters are color-coded by geographic proximity and collaborative intensity.

Among corresponding author affiliations, the United States ranked first with 642 publications, far surpassing China (154), the United Kingdom (105), and Canada (102), which occupied the subsequent positions. A minimum of 47 publications were required to enter the top 10 (**Figure 2A**). The United States has consistently outperformed other countries since the early 2000s. Notably, after 2013, U.S. annual output nearly tripled within a few years and sustained growth to nearly 80 publications per year. Subsequently, U.S. output followed a declining trend similar to the overall field, returning by 2025 to levels observed in 2014-2015. Other major countries exhibited broadly similar trajectories, with overall increases before 2021 followed by declines (**Figure 2A**).

Citation burst analysis identified the United States as having the strongest burst strength (6.77), with peak activity during 2010–2013, followed by Japan (active during 2002–2014). Other countries with notable burst strengths included the United Kingdom, India, and the Netherlands (**Table 1**). These burst patterns were reflected in total citation counts, with the United States accumulating the highest number of citations, followed by China, Canada, and the United Kingdom. Notably, Senegal appeared among the top 10 most-cited countries despite not ranking in the top 10 by publication volume (**Figure 2B**).

**Table 1:** Top 24 countries with the strongest citation bursts.

Countries	Year	Strength	Begin	End	1990 - 2026
UK	1997	5.96	1997	2016	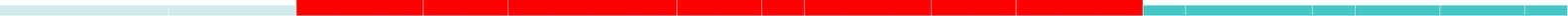
Japan	2002	6.49	2002	2014	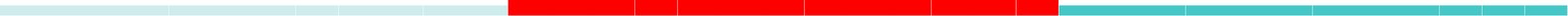
China	2008	1.86	2008	2017	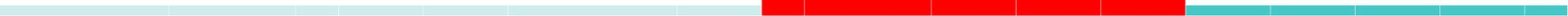
USA	1992	6.77	2010	2013	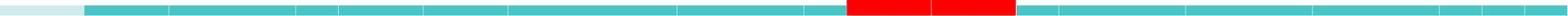
Austria	2010	2.3	2010	2015	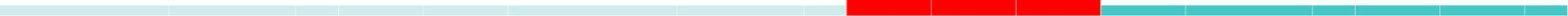
Canada	2005	4.1	2011	2014	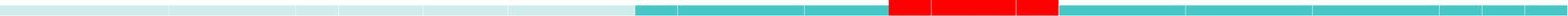
Guinea	2016	3.01	2016	2019	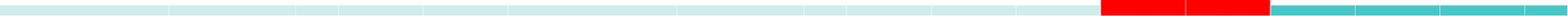
Russia	2013	1.9	2016	2017	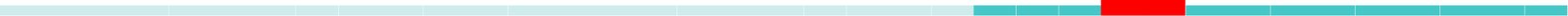
Liberia	2016	2.64	2017	2019	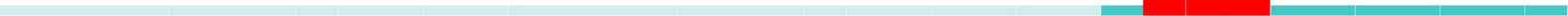
United States	2016	1.91	2018	2022	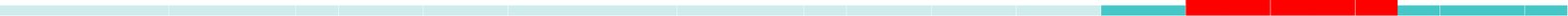
India	2007	5.64	2020	2023	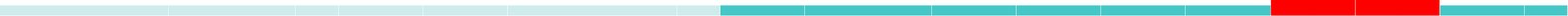
Scotland	2016	3.14	2020	2021	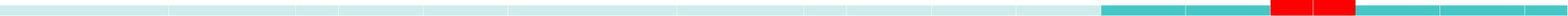
Brazil	2014	3.24	2021	2024	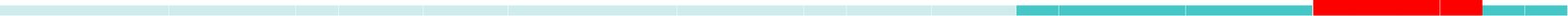
Egypt	2021	2.4	2021	2022	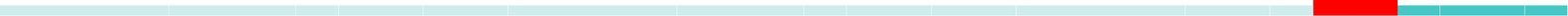
Kenya	2018	2.4	2021	2022	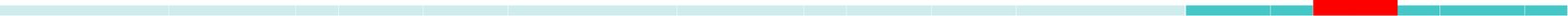
Malaysia	2016	2.52	2022	2026	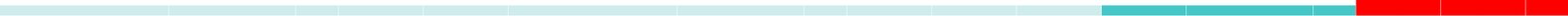
Iran	2015	1.96	2022	2023	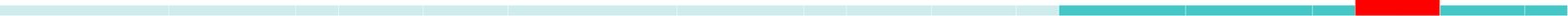
The Netherlands	2016	1.84	2022	2026	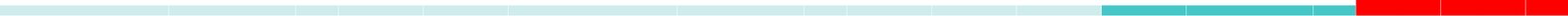
Netherlands	2015	5.24	2023	2024	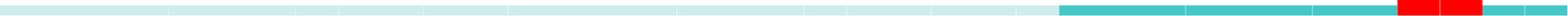
Dem Rep Congo	1999	2.79	2023	2026	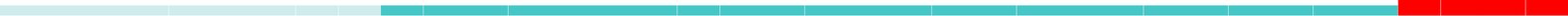
Sweden	2012	2.04	2023	2024	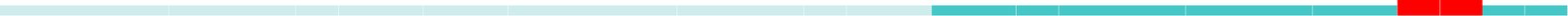
South Africa	2014	1.88	2023	2026	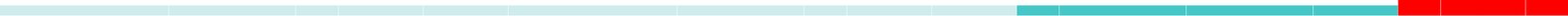
Tanzania	2024	2.25	2024	2026	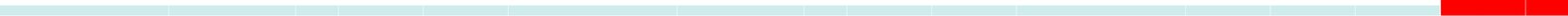
Rep Congo	2022	2.12	2024	2026	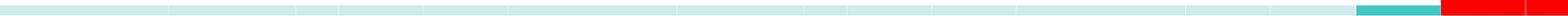

Red bars indicate burst periods; blue shades denote non-burst intervals (light blue: not yet appeared; dark blue: present but no burst).

A two-dimensional plot of the top 10 most productive countries by publication volume and citation impact positioned the United States in the upper-right quadrant, indicating superior performance in both dimensions. China, the United Kingdom, Switzerland, and France were situated in the lower-right quadrant, suggesting relatively higher citation impact per publication, whereas Germany, Italy, and India occupied the lower-left quadrant, indicating greater publication volume relative to citation impact (**Figure 2C**).

International collaboration networks revealed a major cluster comprising North American countries (the United States and Canada), European nations (the United Kingdom, the Netherlands, and Germany), Australia, and South Korea. China also maintained close ties with Asian countries including Pakistan, Iran, and India, which in turn had strong connections to the aforementioned cluster. Additionally, France exhibited notable collaborative links with African nations, including Guinea and the Democratic Republic of the Congo (**Figure 2D**).

### 3.3 Leading institutions

**Figure 3:**
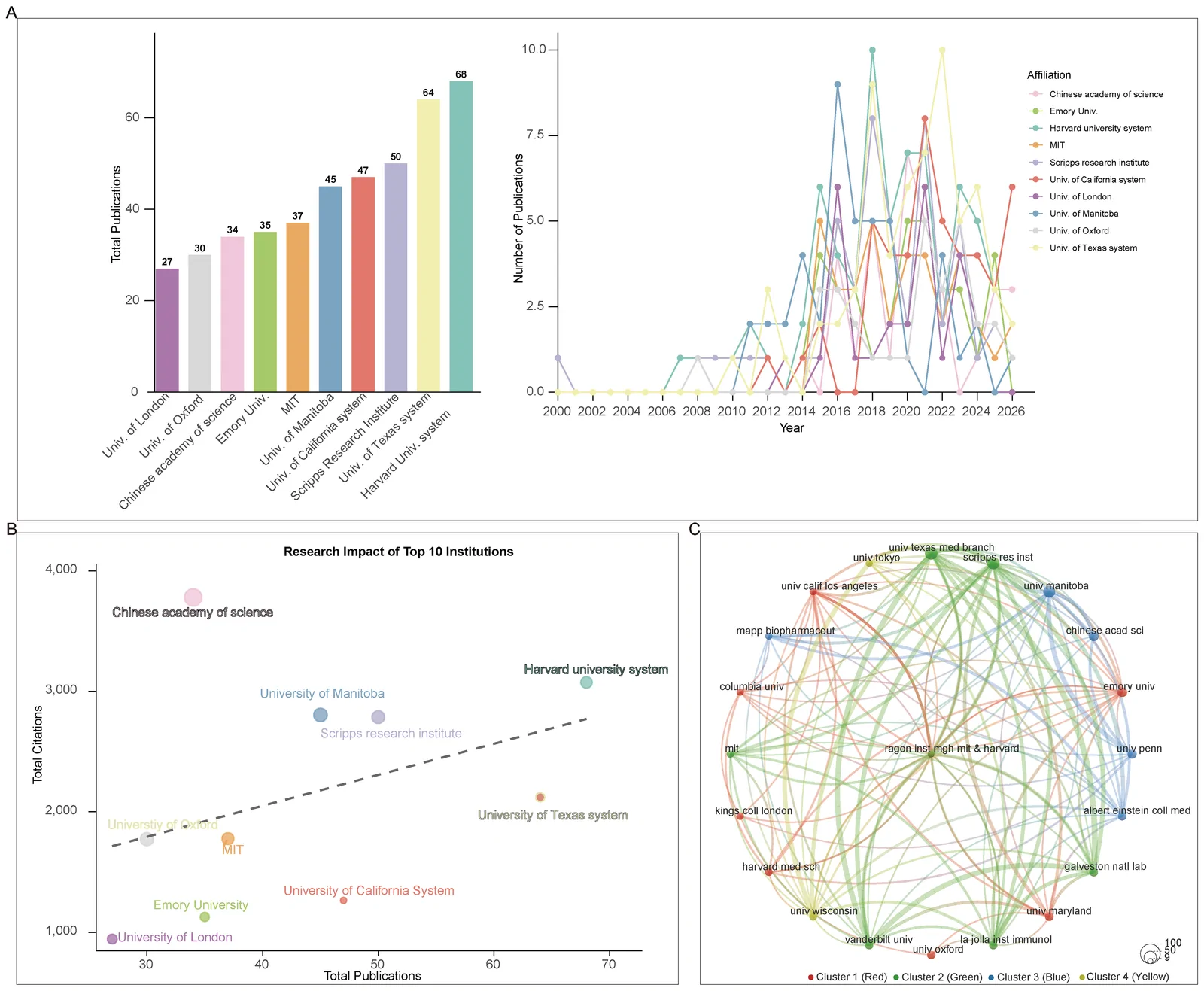
Institutional publication performance and collaboration networks. **(A)** Annual publication output of the top 10 most productive institutions. **(B)** Two-dimensional scatter plot of total publications versus total citations for the top 10 institutions. **(C)** Institutional collaboration network, with node size representing publication volume, edge thickness indicating collaboration strength, and clusters color-coded by modularity class. Major clusters are labeled by their central institutions.

Among high-impact institutions, Harvard University and the University of Texas System ranked first and second with 68 and 64 publications, respectively, followed by Scripps Research Institute (50) and the University of California System (47). A minimum of 28 publications was required to enter the top 10 (**Figure 3A**). Temporal trends at the institutional level mirrored overall publication patterns, with many institutions experiencing rapid growth after 2010, peaking around 2014–2018, followed by a subsequent decline (**Figure 3A**).

Citation burst analysis identified the University of Manitoba as having the strongest burst strength (7.14), followed by the Public Health Agency of Canada, Laval University, and the La Jolla Institute for Immunology (**Table 2**). A two-dimensional scatter plot of total publications versus total citations placed Harvard University in the upper-right quadrant, demonstrating dual leadership. The Chinese Academy of Sciences, the University of Manitoba, and Scripps Research Institute were positioned closer to the citation axis, whereas the University of Texas System, the University of California System, and MIT were situated nearer to the publication axis (**Figure 3B**).

**Table 2:** Top institutions with the strongest citation bursts.

Institutions	Year	Strength	Begin	End	1990 - 2026
Arizona State University	2010	3.27	2010	2015	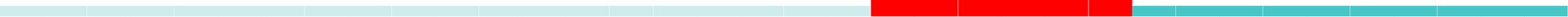
University of Manitoba	2011	7.14	2011	2017	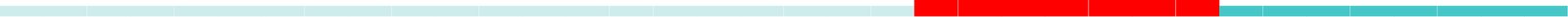
Public Health Agency of Canada	2005	5.89	2011	2017	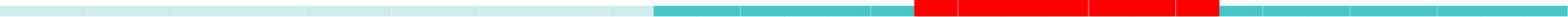
University of Pennsylvania	2006	3.66	2013	2016	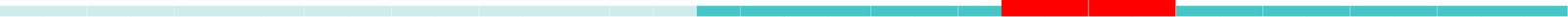
Takara Bio Inc	2014	2.5	2014	2015	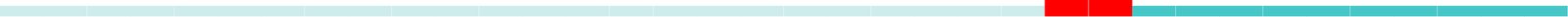
Public Health England	2005	2.49	2015	2016	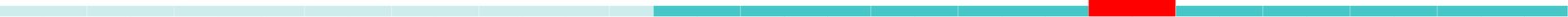
University of Antwerp	2016	3.32	2016	2017	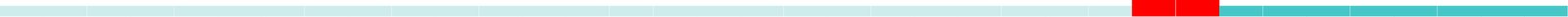
World Health Organization	2016	2.78	2016	2019	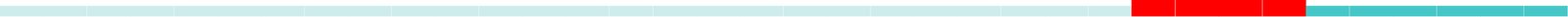
Lomonosov Moscow State University	2016	2.76	2016	2017	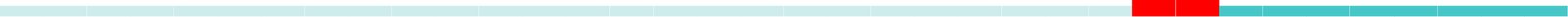
Pasteur Network	2016	2.52	2016	2019	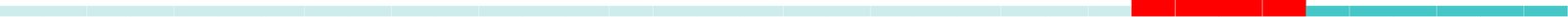
Laval University	2017	5.17	2017	2019	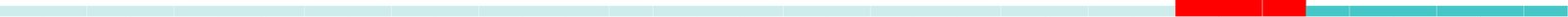
University of Nebraska System	2020	4.26	2020	2024	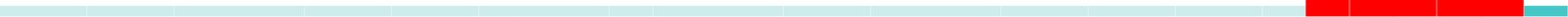
German Center for Infection Research	2018	3.76	2020	2023	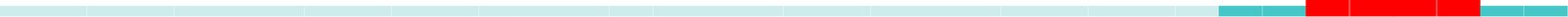
University of Edinburgh	2020	2.46	2020	2021	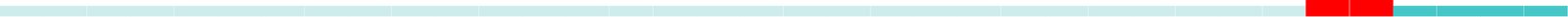
Washington University (WUSTL)	2020	2.46	2020	2021	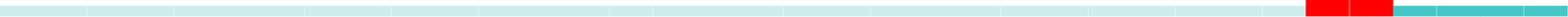
La Jolla Institute for Immunology	2019	4.53	2021	2026	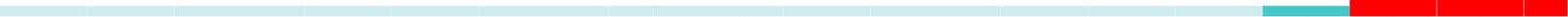
University of Oxford	2008	4.11	2021	2023	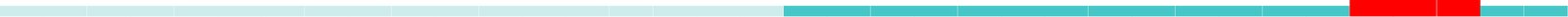
University of California Los Angeles	2018	3.43	2021	2022	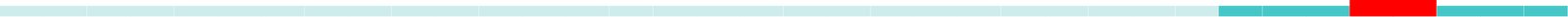
Stanford University	2012	3.67	2022	2026	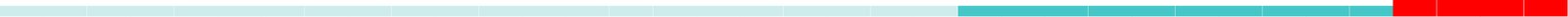
Johns Hopkins University	2022	2.61	2022	2026	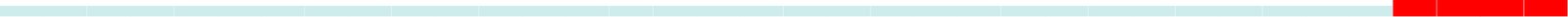
Centers for Disease Control & Prevention - USA	1992	2.5	2022	2024	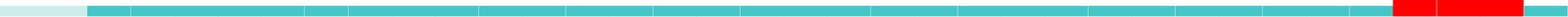
Universite de Kinshasa	2023	4.17	2023	2026	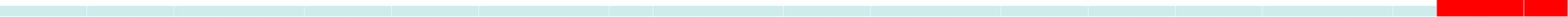

Red bars indicate burst periods; blue shades denote non-burst intervals (light blue: not yet appeared; dark blue: present but no burst).

Institutional collaboration networks formed several distinct clusters. The most prominent red cluster (Cluster 1) included Columbia University, King’s College London, and Emory University. The green cluster (Cluster 2) comprised Harvard University, Scripps Research Institute, the University of Texas System, and MIT. Cluster 3 included Mapp Biopharmaceutical, the University of Manitoba, the Chinese Academy of Sciences, and the University of Pennsylvania. A fourth cluster consisted of the University of Tokyo and the University of Wisconsin, among others (**Figure 3C**).

### 3.4 Core journals

**Figure 4:**
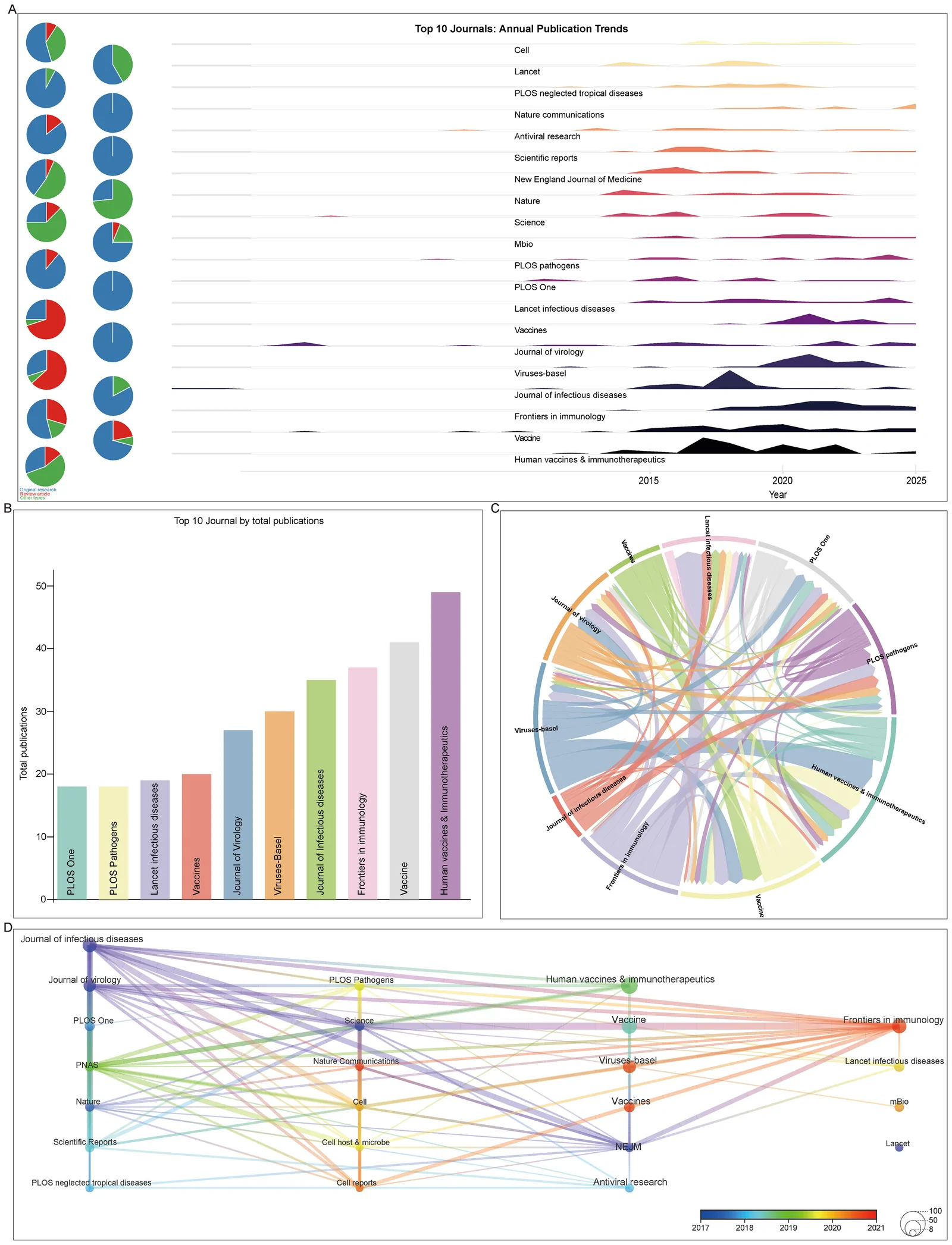
Journal publication patterns, citation relationships, and temporal activity. **(A)** Annual publication output of the top 20 most productive journals, with stacked bars indicating document type composition (original articles, reviews, and others). **(B)** Total citation of top journals. **(C)** Citation relationships among core journals, with node size representing citation count and edge thickness indicating citation strength. **(D)** Journal co-citation network highlighting thematic clusters and temporal activity patterns. The timeline gradient (blue to red) indicates the period of peak activity for each journal, with red indicating more recent activity.

*Human Vaccines & Immunotherapeutics* ranked first in publication volume, predominantly publishing document types other than original research and reviews (e.g., case reports and brief reports), followed by *Vaccine*, *Frontiers in Immunology*, the *Journal of Infectious Diseases*, *Viruses-Basel*, and the *Journal of Virology*. Notably, with the exception of *Viruses-Basel*-where review articles outnumbered original research-all other journals published more original articles than reviews (**Figure 4A**). High-impact journals such as *Nature*, *The Lancet*, and *Science* exhibited publication peaks around 2015, followed by increased output from *Human Vaccines & Immunotherapeutics* and *the Journal of Infectious Diseases* around 2018. After 2020, *Frontiers in Immunology*, *Viruses-Basel*, and *Vaccines* emerged as major contributors (**Figure 4A**).

Total citations largely correlated with publication volume (**Figure 4B**), with *Human Vaccines & Immunotherapeutics* again ranking first, followed by *Vaccine*, *Frontiers in Immunology*, the *Journal of Infectious Diseases*, and *Viruses-Basel*. Citation burst analysis identified *PNAS* as having the strongest burst strength (24.79), followed by the *Journal of Virology* (17.8), *Science Translational Medicine* (17.13), *Nature* (15.43), and *Frontiers in Immunology* (15.35). Early active journals included *PNAS*, the *Journal of Virology*, *Archives of Virology*, and *Virology*, whereas more recently active journals included *Nature Methods*, *EBioMedicin*e, The *Lancet Microbe*, and *Protein Science* (**Table 3**). Citation relationship analysis revealed that *Human Vaccines & Immunotherapeutics* was both heavily citing and widely cited, with strong connections to *Viruses-Basel*, *Vaccine*, and *Frontiers in Immunology*. The journals that most frequently cited *Human Vaccines & Immunotherapeutics* included *PLOS ONE*, the journal itself (self-citation), and The *Lancet Infectious Diseases*. *Vaccine* similarly maintained dense citation relationships with *Vaccines*, *Viruses-Basel*, *Frontiers in Immunology*, and *Human Vaccines & Immunotherapeutics* (**Figure 4C**). *Frontiers in Immunology* emerged as a recently highly active journal with broad citation connections to *Vaccines*, *Viruses-Basel*, and *The Lancet Infectious Diseases*, the latter of which also showed strong ties to *Vaccine*, *PLOS Pathogens*, and the *NEJM*. Notably, core biomedical journals including *Cell*, *Science*, *NEJM*, and *The Lancet* occupied central positions in the temporal activity map, with *NEJM* serving as a particularly important bridging node (**Figure 4C**).

**Table 3:** Top 36 cited journals with the strongest citation bursts.

Cited Journals	Year	Strength	Begin	End	1990 - 2026
P Natl Acad Sci Usa	2009	24.79	2009	2019	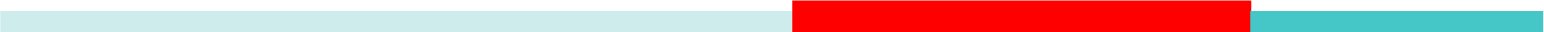
J Virol	2009	17.8	2009	2018	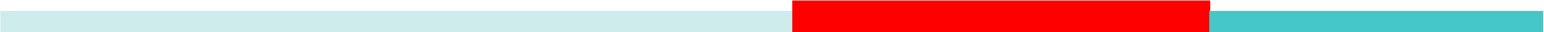
Arch Virol	2009	13.37	2009	2017	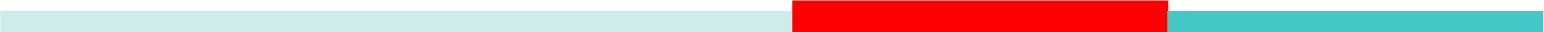
Virology	2009	12.98	2009	2018	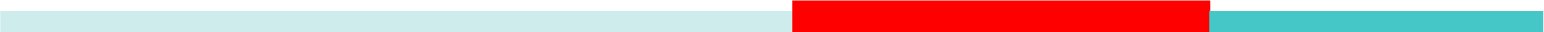
J Infect Dis	2009	10.37	2009	2019	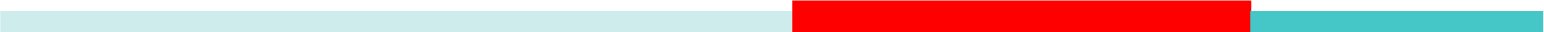
J Gen Virol	2009	7.39	2009	2015	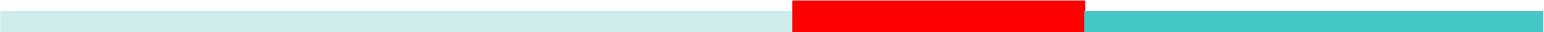
Fields Virology	2009	6.52	2009	2015	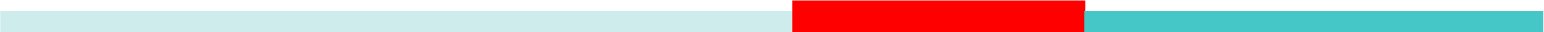
Nature	2010	15.43	2010	2018	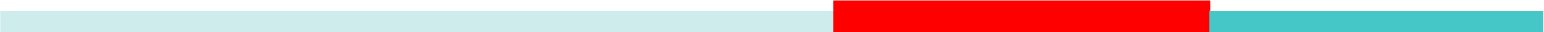
Science	2010	12.76	2010	2019	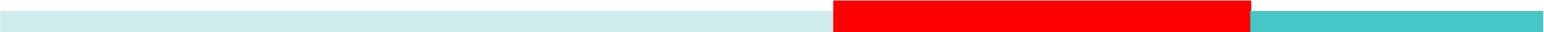
Cancer Res	2010	8.7	2010	2018	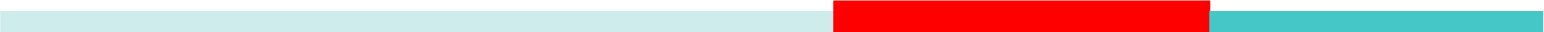
J Biol Chem	2010	6.73	2010	2016	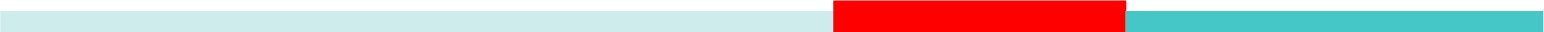
Plos Pathog	2009	12.8	2011	2018	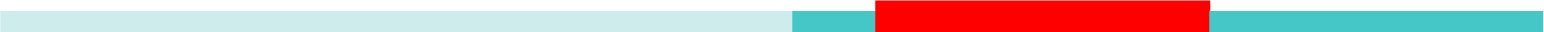
Plant Biotechnol J	2011	7.38	2011	2018	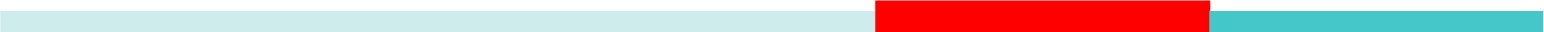
Plos One	2011	13.05	2012	2019	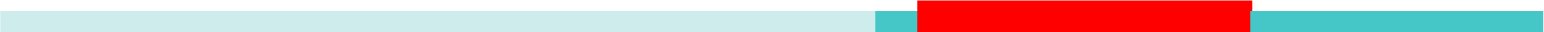
Plos Neglect Trop D	2012	9.31	2012	2018	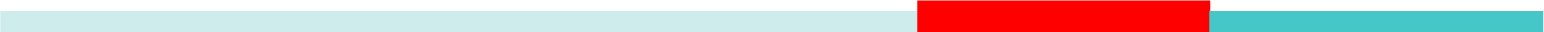
Clin Vaccine Immunol	2012	7.5	2012	2019	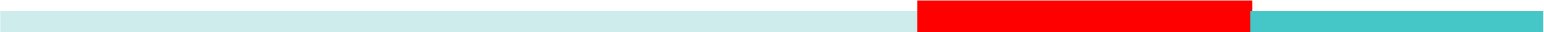
Sci Transl Med	2012	17.13	2013	2019	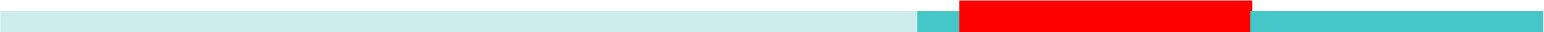
Infect Immun	2013	7.77	2013	2017	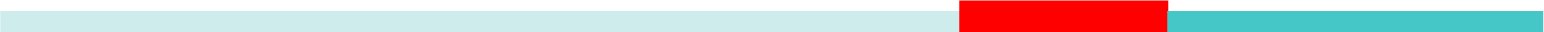
Clin Immunol	2010	7.12	2013	2018	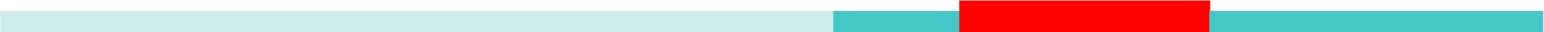
Bmj-Brit Med J	2014	6.88	2014	2017	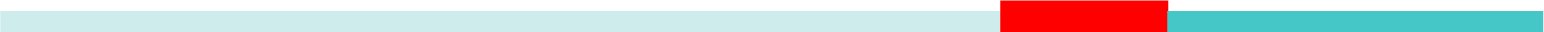
Nat Struct Mol Biol	2012	6.59	2014	2017	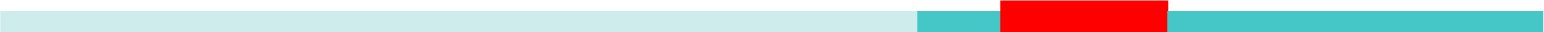
Trends Microbiol	2015	6.76	2015	2018	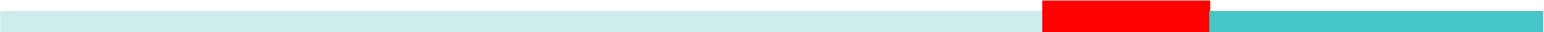
Nat Protoc	2017	6.95	2017	2019	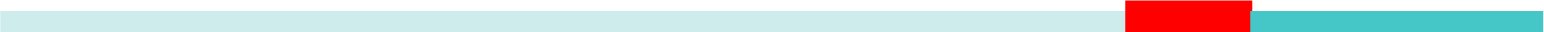
Vaccines-Basel	2019	10.03	2022	2026	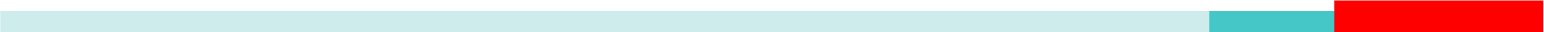
Nat Rev Dis Primers	2022	7.46	2022	2026	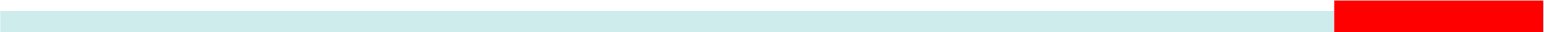
Npj Vaccines	2018	6.79	2022	2026	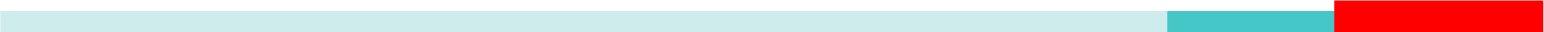
Front Immunol	2017	15.35	2024	2026	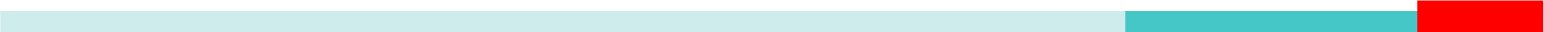
Nat Commun	2016	12.38	2024	2026	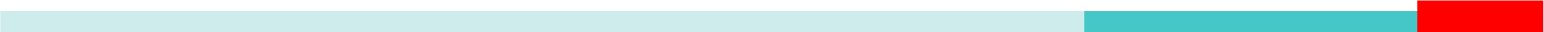
Front Pharmacol	2024	10.23	2024	2026	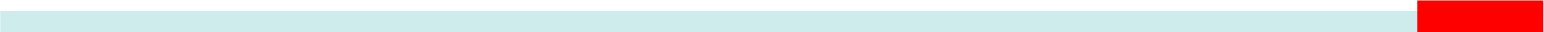
Pathogens	2024	9.15	2024	2026	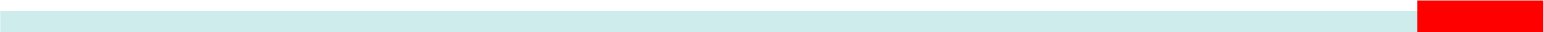
Commun Biol	2024	7.54	2024	2026	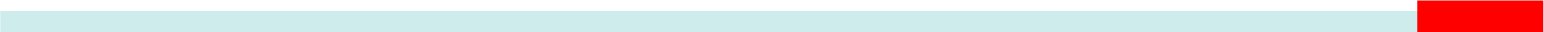
Mol Ther	2013	7.36	2024	2026	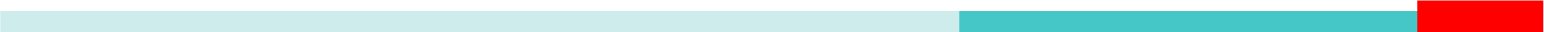
Protein Sci	2022	6.92	2024	2026	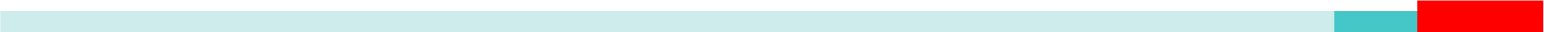
Lancet Microbe	2024	6.83	2024	2026	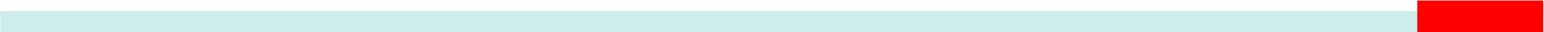
Ebiomedicine	2017	6.74	2024	2026	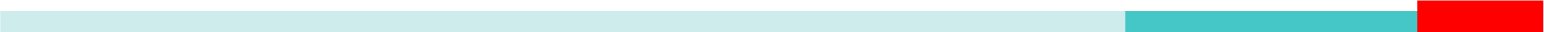
Nat Methods	2018	6.52	2024	2026	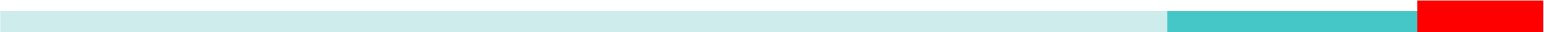

Red bars indicate burst periods; blue shades denote non-burst intervals (light blue: not yet appeared; dark blue: present but no burst).

### 3.5 Influential authors

**Figure 5:**
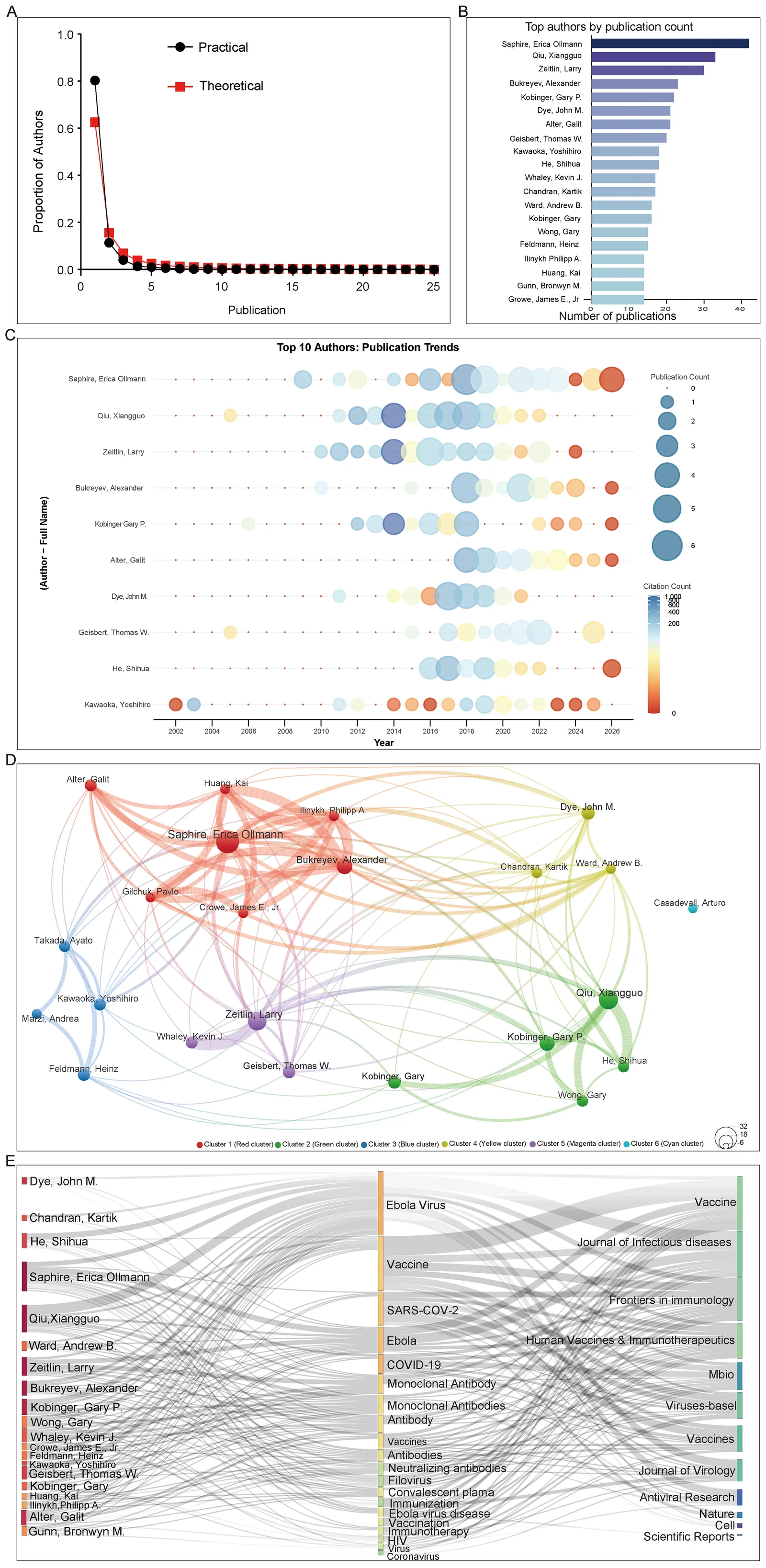
Author productivity, collaboration networks, and thematic associations. **(A)** Author productivity distribution (Lotka’s law), showing the proportion of authors by number of publications. **(B)** Annual publication output of the top 20 most productive authors. **(C)** Annual citation trends for the top 10 most productive authors. **(D)** Author collaboration network, with node size representing publication volume, edge thickness indicating collaboration strength, and clusters color-coded by modularity class. **(E)** Three-field plot illustrating the relationships among key authors (left), author keywords (middle), and core journals (right), with connecting ribbons indicating co-occurrence frequency.

Author productivity analysis, based on Lotka’s law, revealed a highly skewed distribution: the vast majority of authors (>90%) contributed only one or two articles, whereas fewer than 9% published three or more. In stark contrast, only a very small fraction of authors (<3%) published more than five articles, underscoring the exceptional productivity of a tiny minority (**Figure 5A**, **Supplementary Table 2**). The most prolific authors included Dr. Erica Ollmann Saphire (an American structural biologist and immunologist), Dr. Xiangguo Qiu (Director of the Viral Vaccine and Antiviral Therapy Research Unit at the National Microbiology Laboratory, Public Health Agency of Canada), and Dr. Larry Zeitlin, each with over 30 publications. Other highly productive authors included Alexander Bukreyev and Gary P. Kobinger.

Temporal analysis of active years revealed that Yoshihiro Kawaoka was the earliest entrant into the field, with publications dating back to 2002-2003. Thomas W. Geisbert, Xiangguo Qiu, and Gary P. Kobinger began contributing between 2004 and 2006, whereas Erica Ollmann Saphire initiated her high-output phase later but has sustained it to the present. Most authors reached their publication peaks around 2018, with citations peaking for papers published around 2014. Several authors, including Gary P. Kobinger, Larry Zeitlin, and Xiangguo Qiu, accumulated hundreds to thousands of citations. Notably, Xiangguo Qiu and John M. Dye ceased active publication in the field after 2020–2022 (**Figures 5B, C**).

Citation burst analysis identified Xiangguo Qiu as having the strongest burst strength (7.43), followed by Gary P. Kobinger (6.55), Larry Zeitlin (6.37), and the more recently active Placide Mbala-Kingebeni (5.07). In recent years, Steve Ahuka-Mundeke and Daniel Mukadi-Bamuleka have also gained substantial influence (**Table 4**). Collaboration networks grouped influential authors into six clusters, centered respectively around Erica Ollmann Saphire, John M. Dye, Xiangguo Qiu, Larry Zeitlin, Yoshihiro Kawaoka, and Arturo Casadevall (who formed an isolated cluster). Beyond this isolated cluster, extensive cross-cluster collaborations were observed, particularly between the Saphire, Dye, and Zeitlin clusters (**Figure 5D**).

**Table 4:** Top 25 authors with the strongest citation bursts.

Authors	Year	Strength	Begin	End	1990 - 2026
Zeitlin, Larry	2010	6.37	2010	2016	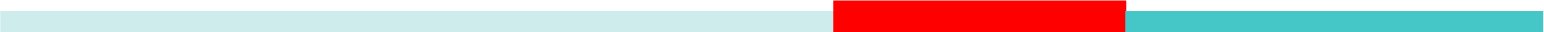
Chen, Qiang	2010	3.31	2010	2016	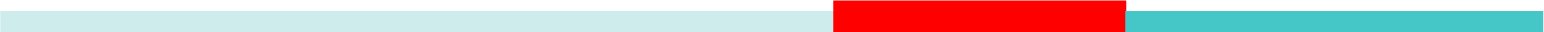
Alimonti, Judie B	2011	3.58	2011	2014	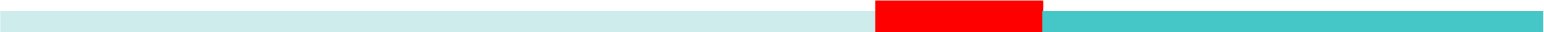
Qiu, Xiangguo	2005	7.43	2012	2019	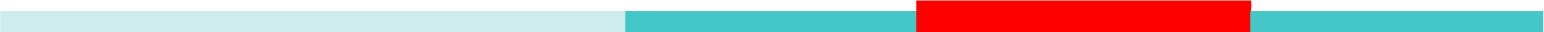
Kobinger, Gary P	2006	6.55	2013	2018	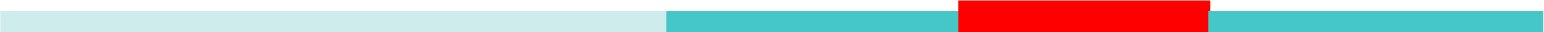
Wong, Gary	2013	3.81	2013	2016	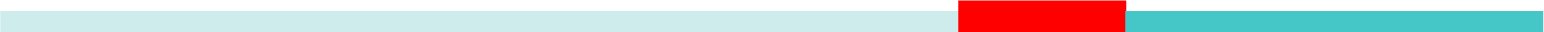
Hiatt, Andrew	2014	3.18	2014	2015	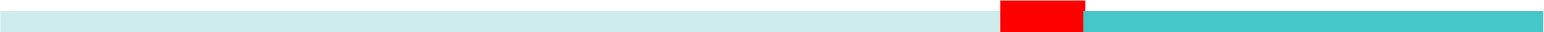
van griensven, Johan	2016	3.73	2016	2016	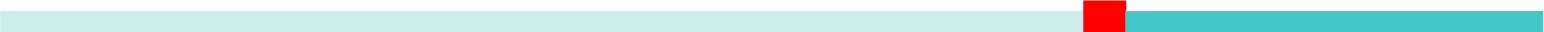
He, Shihua	2016	3.32	2016	2019	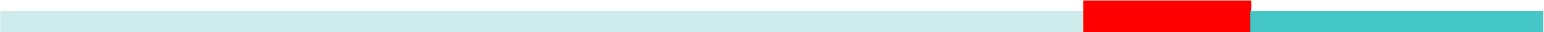
Cagigi, Alberto	2016	3.19	2016	2018	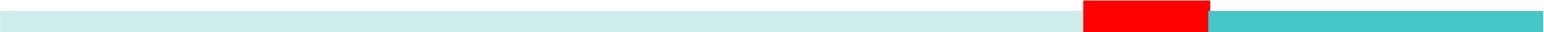
Dye, John M	2011	4.33	2017	2020	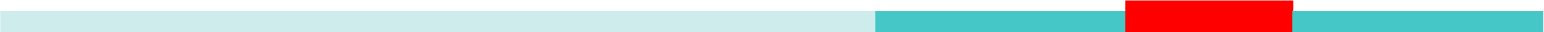
Ward, Andrew B	2017	4	2017	2021	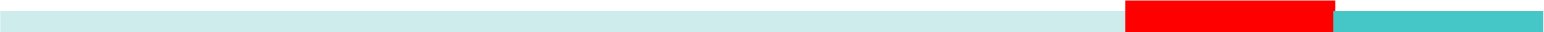
Turner, Hannah L	2017	3.18	2017	2018	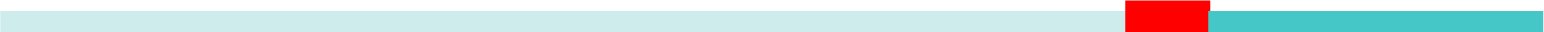
Gunn, Bronwyn M	2018	4.79	2018	2021	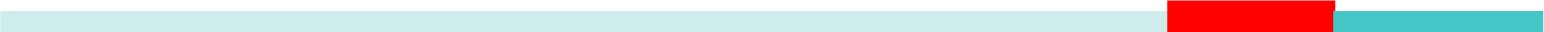
Crowe, James E	2018	4.33	2018	2022	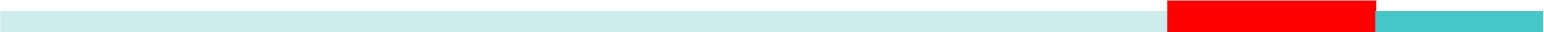
Alter, Galit	2018	4.32	2018	2023	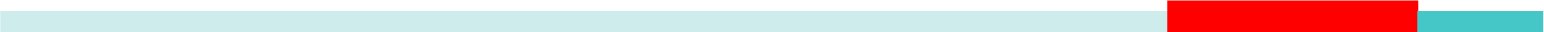
Ilinykh, Philipp A	2018	3.98	2018	2021	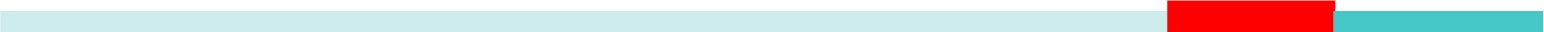
Huang, Kai	2018	3.98	2018	2021	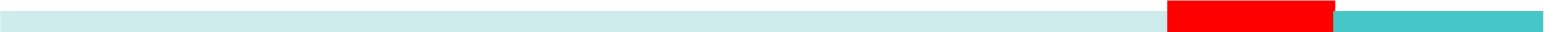
Bukreyev, Alexander	2010	3.52	2018	2022	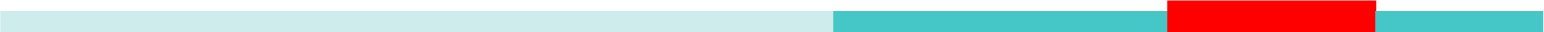
Gilchuk, Pavlo	2018	3.18	2018	2021	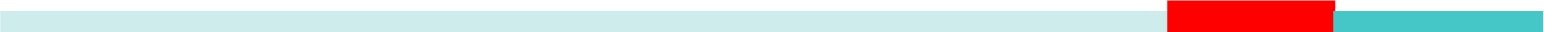
Geisbert, Thomas W	2005	3.3	2020	2022	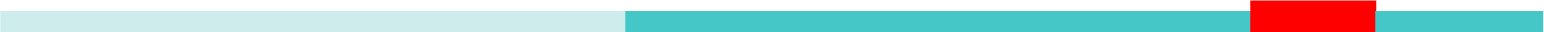
Douoguih, Macaya	2021	3.63	2021	2024	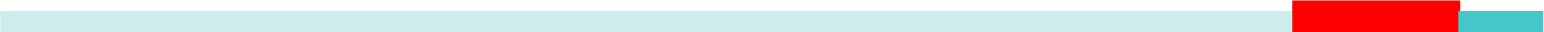
Mbala-kingebeni, Placide	2019	5.07	2023	2026	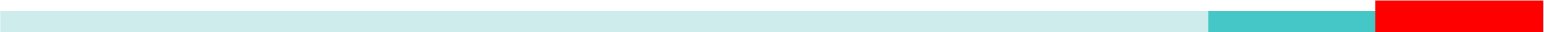
Ahuka-mundeke, Steve	2023	3.76	2023	2026	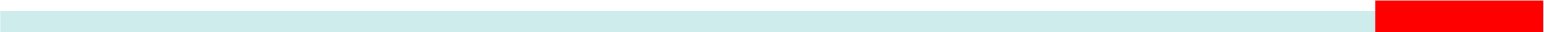
Mukadi-bamuleka, Daniel	2024	4.1	2024	2026	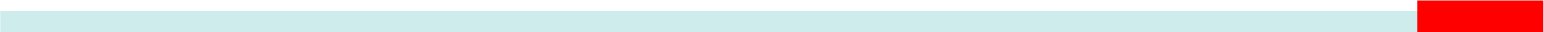

Red bars indicate burst periods; blue shades denote non-burst intervals (light blue: not yet appeared; dark blue: present but no burst).

The three-field plot (authors, keywords, journals) revealed that many authors bridged multiple research domains. For example, Erica Ollmann Saphire was involved not only in Ebola and antibody research but also in COVID-19 (SARS-CoV-2) and filovirus studies. Similarly, high-impact authors including Bronwyn M. Gunn and Galit Alter were also engaged in COVID-19 research. Additionally, a subset of authors, including Gary P. Kobinger and Kevin J. Whaley, contributed to HIV research. Key terms such as “Ebola virus,” “vaccine,” and “antibody” appeared consistently across all core journals (**Figure 5E**).

### 3.6 Research frontiers and evolving trends

**Figure 6:**
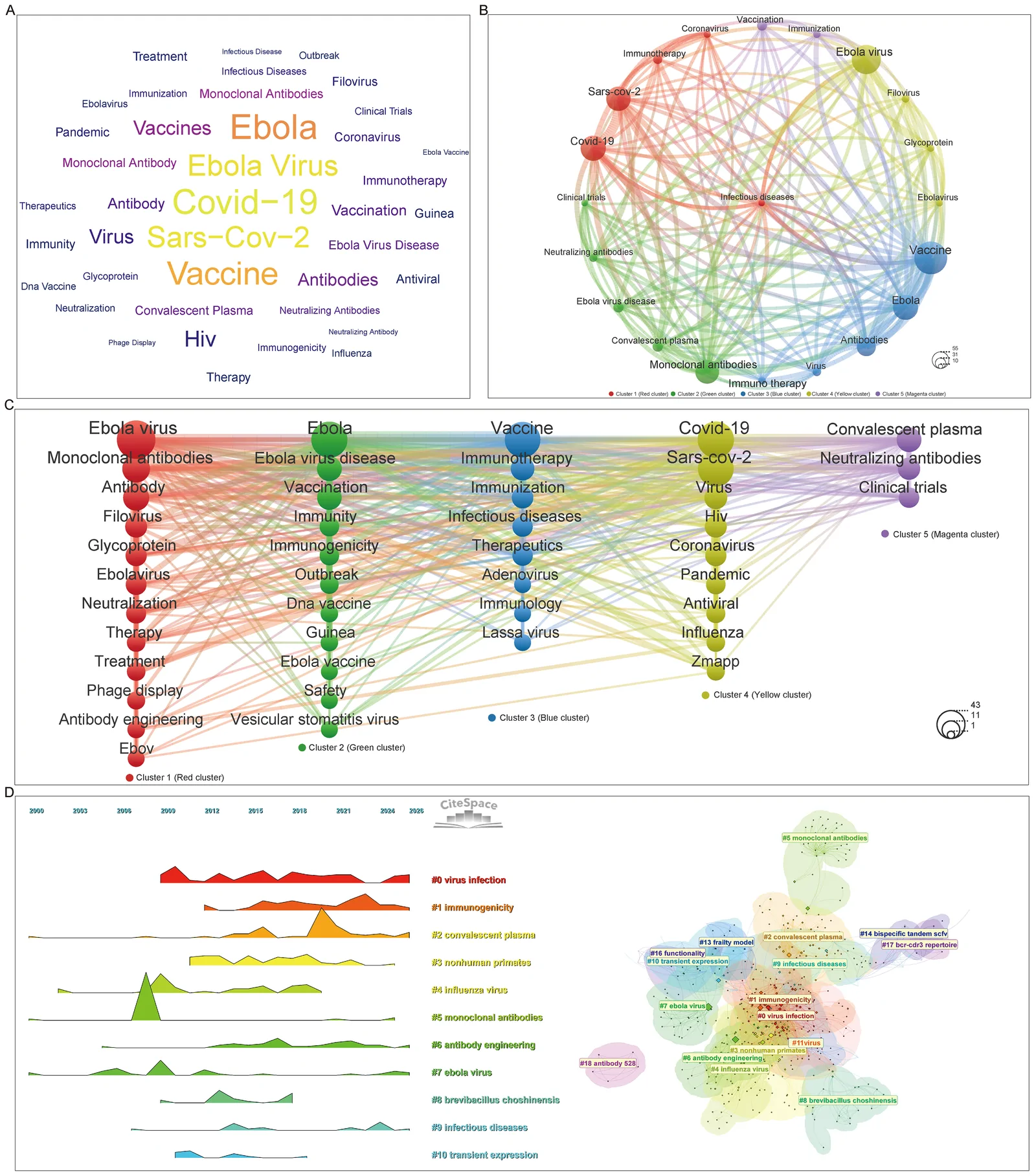
Keyword co-occurrence networks and thematic clusters. **(A)** Word cloud of high-frequency keywords, with font size proportional to occurrence frequency. **(B)** Keyword co-occurrence network, with node size representing frequency and edge thickness indicating co-occurrence strength. Clusters are color-coded by thematic grouping. **(C)** VOSviewer-generated keyword clustering map, with each cluster color-coded and labeled by its predominant theme. **(D)** CiteSpace-generated burst detection and timeline visualization of keyword clusters.

Keyword analysis revealed that terms with frequencies comparable to “Ebola” and “Ebola virus” included “COVID-19,” “SARS-CoV-2,” “vaccine,” “vaccines,” “antibody,” “antibodies,” and “monoclonal antibody.” Moderately frequent terms included “HIV,” “antiviral,” and “filovirus” (**Figure 6A**). Cluster analysis categorized keywords into five thematic groups. Cluster 1 centered on COVID-19 and SARS-CoV-2; Cluster 2 on Ebola virus, filovirus, and glycoprotein; Cluster 3 on Ebola virus disease, neutralizing antibodies, and clinical trials; Cluster 4 on Ebola, vaccine, and antibodies; and Cluster 5 on vaccination and immunization. These clusters exhibited substantial interconnections, with “infectious disease” serving as a central bridge node (**Figure 6B**).

VOSviewer clustering of mid- to high-frequency keywords further refined these groupings. The red cluster (Ebola virus) encompassed fundamental virology (filovirus, EBOV) and antibody engineering (glycoprotein, antibody phage display, antibody engineering). The second cluster focused on immunity and therapy (vaccination, immunogenicity, safety). The third cluster addressed infection and treatment (infectious disease, therapeutics, immunization). The fourth cluster connected multiple viruses (COVID-19, HIV, coronavirus, influenza, ZMapp). The fifth cluster comprised clinically oriented terms (clinical trials, neutralizing antibodies, convalescent plasma) (**Figure 6C**).

Temporal analysis of frontier topics revealed that the earliest active area was fundamental virology (Ebolavirus), followed by convalescent plasma and subsequently antibody-focused research (monoclonal antibodies, antibody engineering). More recently active topics included virus infection, immunogenicity, and infectious diseases. Transiently active topics included Brevibacillus choshinensis, influenza virus, and nonhuman primates. CiteSpace burst detection corroborated these findings, with immunogenicity and virus infection emerging as core research foci (**Figure 6D**).

**Figure 7:**
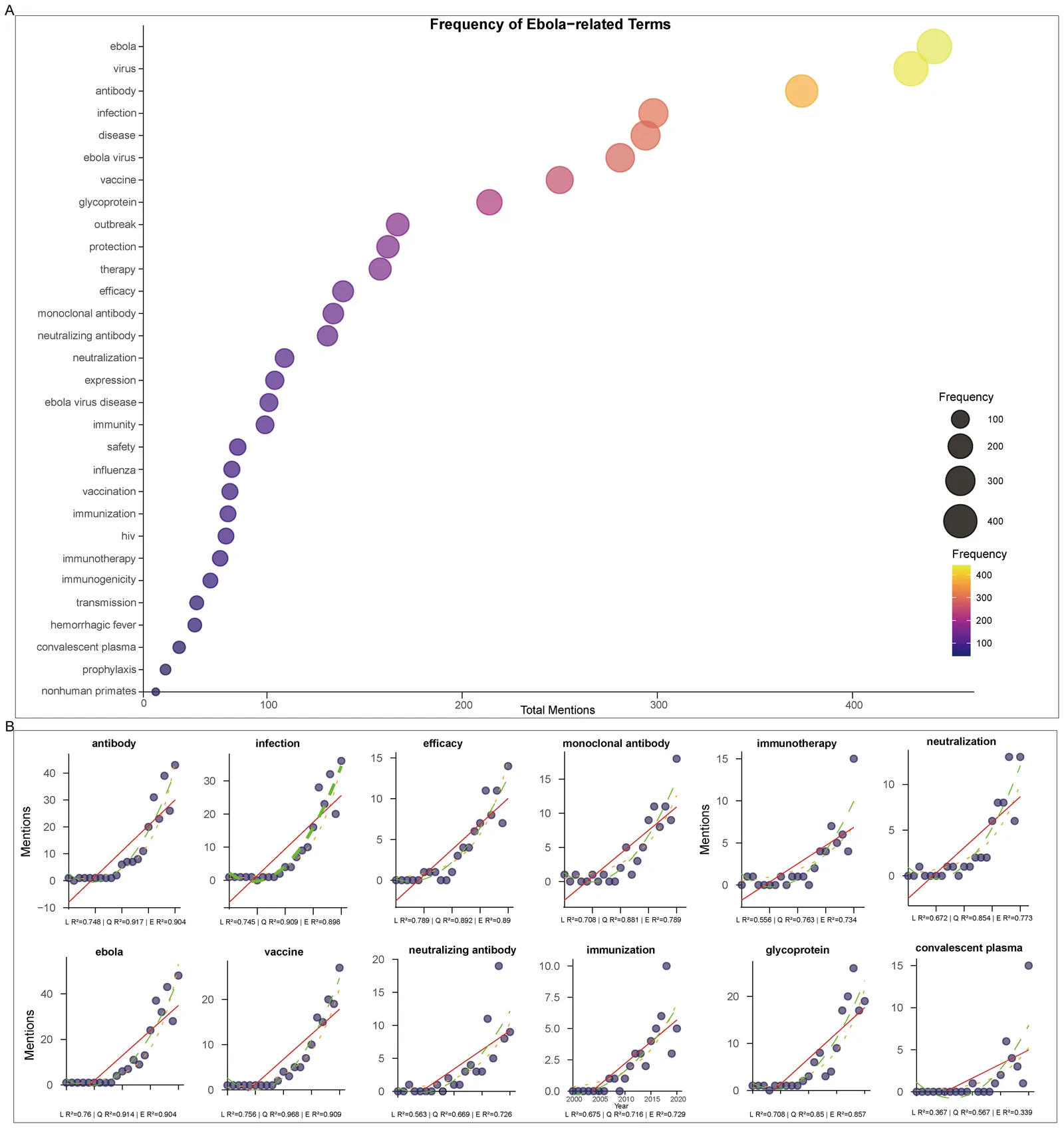
Temporal trends of high-frequency keywords. **(A)** Total frequency of representative high-frequency keywords. **(B)** Comparison of selected keywords (e.g., antibody, infection, efficacy) exhibiting near-exponential growth before 2020 followed by post-2020 deceleration.

Frequency analysis of high-occurrence keywords showed that Ebola-related terms (Ebola virus, Ebola virus disease) and antibody-related terms (monoclonal antibody, neutralizing antibody) appeared most frequently, alongside infection-related terms (infection, outbreak, transmission, prophylaxis) and virological terms (glycoprotein). Temporal trend analysis revealed that several keywords-including antibody, infection, and efficacy-exhibited classic exponential growth from 2000 to 2020, followed by a marked deceleration after 2021, mirroring the overall publication trend (**Figures 7A, B**).

Based on the citation burst analysis (**Table 5**), we further examined the top papers that have shaped the Ebola research landscape over the past decades. The most prominent bursts occurred around 2014–2016, coinciding with the West African Ebola epidemic. Notably, the randomized controlled trial of ZMapp (Qiu et al., Nature, 2014) (32) exhibited the highest burst strength (25.29), reflecting its seminal impact on therapeutic development. Other highly bursty papers include early proof-of-concept studies on post-exposure prophylaxis using monoclonal antibodies (e.g., Dye et al., PNAS, 2012; Pettitt et al., Sci Transl Med, 2013) (33, 34) and the subsequent clinical evaluation of ZMapp (NEJM, 2016) (35). More recent bursts (2021–2024) are associated with large-scale therapeutic trials and review articles, such as the PALM trial (NEJM, 2019) (36) and a comprehensive Lancet review on Ebola virus disease. Of note, the latest burst (2024–2026) features the FDA-approved drug Ebanga™ (Front Pharmacol, 2023) (37), indicating a shift from outbreak-response research to regulatory and clinical translation. Collectively, these highly cited papers reveal a clear trajectory from basic antibody discovery and animal model validation, through controlled clinical trials, to the eventual licensing of specific therapeutics-demonstrating the maturation of the Ebola countermeasure field.

**Table 5:** Top 25 publications with the strongest citation bursts.

Title	Journal	Year	Strength	Begin	End	1990 - 2026
Postexposure antibody prophylaxis protects nonhuman primates from filovirus disease	PNAS	2012	13.82	2012	2017	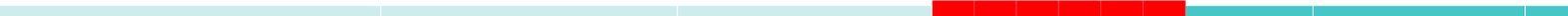
Postexposure protection of non-human primates against a lethal Ebola virus challenge with RNA interference: a proof-of-concept study	Lancet	2010	8.05	2012	2015	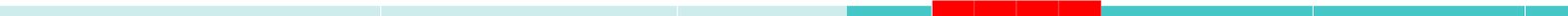
Delayed treatment of Ebola virus infection with plant-derived monoclonal antibodies provides protection in rhesus macaques	PNAS	2012	17.1	2013	2017	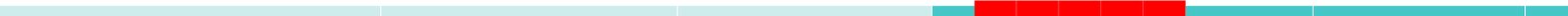
Successful Treatment of Ebola Virus–Infected Cynomolgus Macaques with Monoclonal Antibodies	Sci Transl Med	2012	16.66	2013	2017	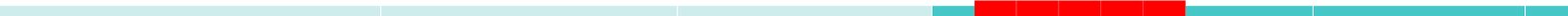
Protective Efficacy of Neutralizing Monoclonal Antibodies in a Nonhuman Primate Model of Ebola Hemorrhagic Fever	Plos One	2012	7.68	2013	2016	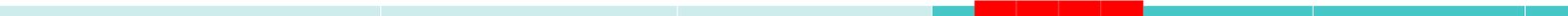
Ebola GP-Specific Monoclonal Antibodies Protect Mice and Guinea Pigs from Lethal Ebola Virus Infection	Plos Neglect Trop D	2012	7.63	2013	2017	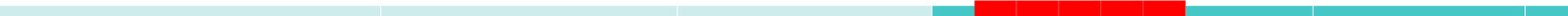
Characterization of Zaire ebolavirus glycoprotein-specific monoclonal antibodies	Clin Immunol	2011	7.17	2013	2016	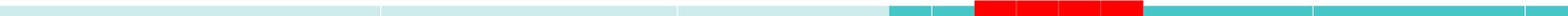
Therapeutic Intervention of Ebola Virus Infection in Rhesus Macaques with the MB-003 Monoclonal Antibody Cocktail	Sci Transl Med	2013	10.5	2014	2018	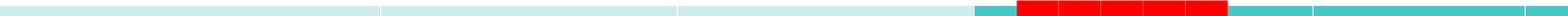
Reversion of advanced Ebola virus disease in nonhuman primates with ZMapp	Nature	2014	25.29	2015	2019	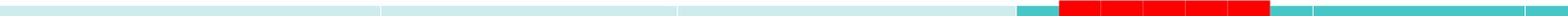
Structures of protective antibodies reveal sites of vulnerability on Ebola virus	PNAS	2014	12.17	2015	2019	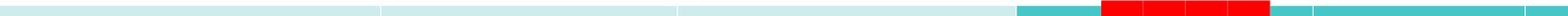
Molecular Characterization of the Monoclonal Antibodies Composing ZMAb: A Protective Cocktail Against Ebola Virus	Sci Rep	2014	8.02	2016	2019	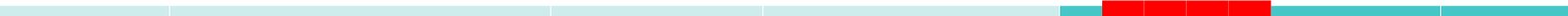
Mechanism of Binding to Ebola Virus Glycoprotein by the ZMapp, ZMAb, and MB-003 Cocktail Antibodies	J Virol	2015	6.63	2016	2019	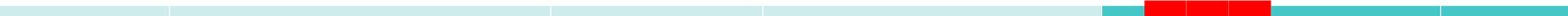
A Randomized, Controlled Trial of ZMapp for Ebola Virus Infection	NEJM	2016	10.41	2017	2019	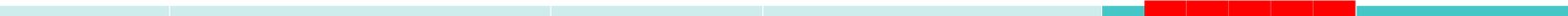
Protective monotherapy against lethal Ebola virus infection by a potently neutralizing antibody	Science	2016	9.16	2017	2021	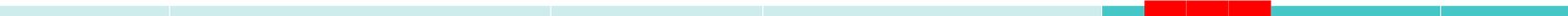
Isolation of potent neutralizing antibodies from a survivor of the 2014 Ebola virus outbreak	Science	2016	8.07	2017	2019	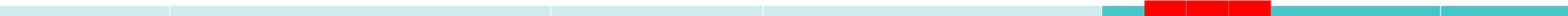
Cross-Reactive and Potent Neutralizing Antibody Responses in Human Survivors of Natural Ebolavirus Infection	Cell	2016	7.14	2017	2019	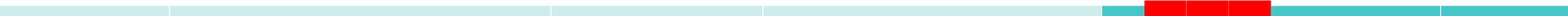
Structural and molecular basis for Ebola virus neutralization by protective human antibodies	Science	2016	7.14	2017	2019	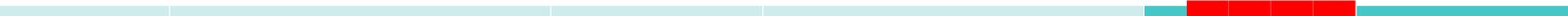
Antibodies from a Human Survivor Define Sites of Vulnerability for Broad Protection against Ebolaviruses	Cell	2017	6.85	2018	2021	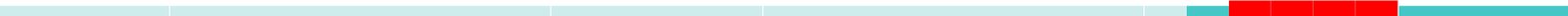
Development of Clinical-Stage Human Monoclonal Antibodies That Treat Advanced Ebola Virus Disease in Nonhuman Primates	J Infect Dis	2018	8.62	2019	2022	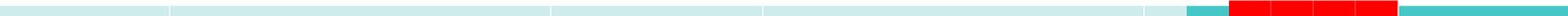
Systematic Analysis of Monoclonal Antibodies against Ebola Virus GP Defines Features that Contribute to Protection	Cell	2018	6.82	2019	2022	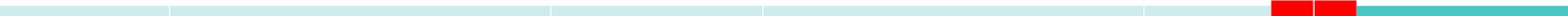
Anticancer kinase inhibitors impair intracellular viral trafficking and exert broad-spectrum antiviral effects	NEJM	2020	7.39	2020	2021	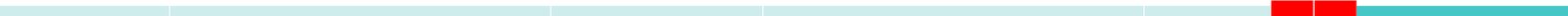
Treatment of 5 Critically Ill Patients With COVID-19 With Convalescent Plasma	Jama	2020	6.95	2020	2021	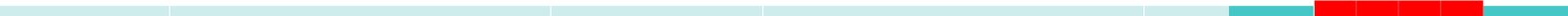
A Randomized, Controlled Trial of Ebola Virus Disease Therapeutics	NEJM	2019	16.38	2021	2024	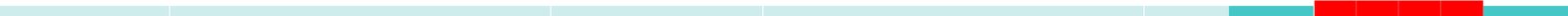
Ebola virus disease	Lancet	2019	7.07	2021	2024	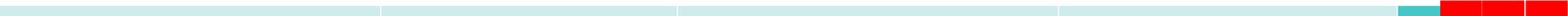
Ebanga™: The most recent FDA-approved drug for treating Ebola	Front Pharmacol	2023	7.79	2024	2026	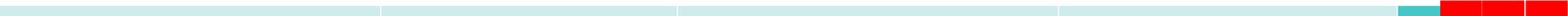

Red bars indicate burst periods; blue shades denote non-burst intervals (light blue: not yet appeared; dark blue: present but no burst).

## 4 Discussion

Our bibliometric study provides a comprehensive overview of the evolution of Ebola antibody therapy research over the past three decades. Rather than representing a steady linear progression, the field has developed in response to major outbreak events, advances in antibody engineering technologies, and changing global public health priorities. Our findings indicate that Ebola antibody therapy research is characterized by three overarching features: outbreak-driven growth, highly concentrated scientific leadership, and a gradual transition from fundamental virology toward translational and clinically oriented research.

### 4.1 Publication trends and their relationship to outbreak dynamics

The exponential growth in Ebola antibody therapy research prior to 2020, followed by a post-peak decline, closely mirrors the temporal pattern of major Ebola outbreaks and the broader infectious disease research landscape. The first major publication peak during 2012–2016 corresponds precisely to the 2014–2016 West African Ebola epidemic-the largest and most devastating in history-which catalyzed unprecedented global research investment and accelerated the development of monoclonal antibody therapies such as ZMapp, mAb114, and REGN-EB3 (15, 19). This outbreak fundamentally transformed the field, shifting Ebola from a neglected tropical disease to a global health priority and demonstrating that antibody-based interventions could significantly reduce mortality in real-world outbreak settings.

The subsequent decline in publications after 2020, despite the ongoing need for Ebola therapeutics, may reflect several interconnected factors. First, the emergence of the COVID-19 pandemic in early 2020 diverted global research attention, funding, and infrastructure toward SARS-CoV-2, temporarily overshadowing Ebola research. Second, the successful licensure of monoclonal antibody treatments (mAb114/Ebanga and REGN-EB3/Inmazeb) for Zaire ebolavirus may have created a perception that the “Ebola problem” had been largely solved, reducing the urgency for further research. Third, the absence of major Zaire ebolavirus outbreaks between 2020 and 2025 likely diminished the immediate impetus for continued investment. However, the emergence of the 2026 Bundibugyo virus outbreak-for which no licensed vaccines or therapeutics exist-has dramatically reignited research urgency, as reflected in renewed Google Trends interest and the WHO’s declaration of a Public Health Emergency of International Concern on 17 May 2026.

This pattern highlights a fundamental challenge for Ebola research: the field operates in a cycle of crisis-driven funding and attention, with periods of intense activity during outbreaks followed by relative quiescence. The current Bundibugyo outbreak starkly illustrates the consequences of this cycle-despite over three decades of research, the world remains unprepared for Ebola strains other than Zaire. Our findings suggest that sustained, outbreak-independent research investment is needed to develop broadly protective antibody therapies effective against all ebolavirus species, rather than relying on reactive responses to individual outbreaks.

### 4.2 Geographic and institutional leadership

The overwhelming dominance of the United States in Ebola antibody therapy far surpassing China, the United Kingdom, and Canada-reflects several structural factors. The U.S. has historically been the primary funder of Ebola research through agencies such as the National Institutes of Health, the Biomedical Advanced Research and Development Authority, and the Department of Defense, which have invested heavily in biodefense-related research. The concentration of leading research institutions-Harvard University, the University of Texas System, Scripps Research Institute, and the University of California System-within the U.S. has created a critical mass of expertise and infrastructure. Furthermore, the U.S. has played a central role in the clinical development of Ebola antibody therapies, including the PALM trial in the DRC that established the efficacy of mAb114 and REGN-EB3.

The emergence of China as the second most productive country reflects its growing investment in global health research and its recent engagement in Ebola response efforts, including the deployment of a multidisciplinary expert team to the DRC. However, the substantial gap between the U.S. and China in both publication volume and citation impact suggests that China’s contribution, while growing, has not yet achieved the same level of influence. The presence of Senegal among the top 10 most-cited countries, despite not ranking in the top 10 by publication volume, highlights the importance of African institutions in providing critical context and data for Ebola research, particularly through outbreak response and clinical trial participation.

Collaboration network analysis reveals a highly interconnected global research community, with the U.S. serving as the central hub. The dense collaborations between North American, European, and Asian institutions reflect the global nature of Ebola research and the shared commitment to addressing this transnational threat. Notably, France’s close collaborative links with Guinea and the Democratic Republic of the Congo reflect historical ties and the presence of French research institutions in West and Central Africa. These collaborations are essential for ensuring that research is informed by local epidemiological realities and that therapeutic advances reach affected populations.

### 4.3 Thematic evolution and research frontiers

The identification of five major research clusters through keyword analysis reveals the intellectual structure of Ebola antibody therapy research. The red cluster (Ebola virus, filovirus, glycoprotein) represents the foundational virology that has underpinned the field since its inception. The glycoprotein, as the sole target of neutralizing antibodies, has been the focus of extensive structural and functional studies, enabling the rational design of antibody therapeutics (14, 38). The antibody engineering cluster (antibody phage display, antibody engineering) reflects the technological advances that have enabled the development of highly potent monoclonal antibodies, including the use of advanced screening and optimization techniques (39, 40).

The second cluster (vaccination, immunogenicity, safety) encompasses the interface between antibody therapy and vaccine development, reflecting the complementary roles of these approaches in Ebola prevention and treatment. The third cluster (infectious disease, therapeutics, immunization) addresses the clinical translation of antibody therapies, including the challenges of delivering these interventions in resource-limited outbreak settings. The fourth cluster’s connection to COVID-19, HIV, coronavirus, and influenza reflects the broader relevance of antibody platform technologies beyond Ebola, as well as the cross-fertilization of ideas and methods across infectious disease research. The fifth cluster (clinical trials, neutralizing antibodies, convalescent plasma) captures the clinical evaluation of antibody therapies, including the use of convalescent plasma as an early therapeutic approach.

The temporal analysis of keyword emergence reveals a clear progression from fundamental virology to translational research. The earliest active area was Ebolavirus, followed by convalescent plasma, and subsequently antibody-focused research including monoclonal antibodies and antibody engineering. More recently active topics include virus infection, immunogenicity, and infectious diseases, reflecting the field’s maturation toward clinically relevant questions.

### 4.4 Immunological insights from the bibliometric trends

The bibliometric trends identified in this study provide a window into the evolving understanding of Ebola antibody immunity. The chronological progression of research topics, from early virological characterization to the current emphasis on antibody engineering and clinical translation, mirrors the field’s deepening immunological knowledge.

The early focus on viral glycoprotein (GP) reflects the fundamental immunological insight that GP is the sole target of neutralizing antibodies against Ebola virus (**Figure 6**, **Figure 7**). Studies identifying the receptor-binding site (RBS) (41), the mucin-like domain (MLD) (42), and the glycan cap (43) as key epitopes have laid the groundwork for rational antibody design. The subsequent rise of monoclonal antibody research corresponds to the discovery that potent neutralizing antibodies can be isolated from human survivors (44), leading to the identification of protective antibody classes such as those targeting the GP base, the RBS, and the fusion loop.

The emergence of antibody engineering as a distinct research cluster (**Figure 6**) highlights the immunological challenges that have driven technological innovation. The need to enhance antibody half-life, improve neutralization potency, and achieve cross-reactivity across ebolavirus species has spurred the development of advanced screening and optimization platforms, including phage display, yeast display, and next-generation sequencing-based approaches (45). These technologies have enabled the identification of broadly neutralizing antibodies such as MBP134, which targets a conserved epitope across multiple ebolavirus species (46, 47).

The recent emphasis on immunogenicity and safety reflects the field’s maturation toward clinically relevant questions. As antibody therapies enter clinical trials, understanding the host immune response to these biologics, including infusion-related reactions, has become increasingly important (46). The focus on combination antibody regimens arises from the immunological principle that targeting multiple epitopes may mitigate viral escape and enhance therapeutic efficacy, particularly in the context of emerging viral variants.

Looking forward, the current Bundibugyo outbreak underscores several pressing immunological questions. First, the protective correlates of immunity for non-Zaire ebolaviruses remain poorly defined, and the extent to which Zaire-specific antibodies cross-react with Bundibugyo virus GP is largely unknown. Second, the development of pan-ebolavirus antibodies targeting conserved epitopes, such as the GP fusion loop or the internal fusion peptide, represents a critical research priority. Third, optimizing Fc-mediated effector functions, including antibody-dependent cellular cytotoxicity (ADCC) (20, 48), may enhance *in vivo* protective efficacy beyond neutralization alone. Finally, the evaluation of combination of antibody regimens and bispecific antibody formats will be essential to address viral diversity and prevent escape. Addressing these immunological challenges will require sustained research investment and international collaboration, ensuring that future outbreaks are met with effective, broadly protective countermeasures.

### 4.5 Clinical translation and the current outbreak

The clinical translation of Ebola antibody therapies represents one of the most significant achievements in the field. The PALM trial demonstrated that mAb114 and REGN-EB3 could reduce mortality when administered early in the course of infection (49, 50). These therapies have since been licensed for the treatment of Zaire ebolavirus, marking a transformative shift from Ebola being a disease with limited therapeutic options to one with effective medical countermeasures.

However, the current Bundibugyo outbreak has exposed a critical vulnerability: the licensed antibody therapies are specific to Zaire ebolavirus and do not protect against other ebolavirus species (51, 52). The WHO has convened expert groups to identify promising candidate therapeutics for Bundibugyo virus disease, prioritizing the monoclonal antibodies MBP134 and Maftivimab, as well as the antiviral remdesivir (WHO). MBP134 is a particularly promising pan-ebolavirus antibody that has demonstrated broad neutralizing activity across multiple species (53, 54). However, these candidates remain investigational and must be evaluated within clinical trials before they can be deployed for widespread use.

The absence of licensed therapeutics for Bundibugyo virus underscores the need for continued research investment in broadly protective antibody therapies. The development of pan-ebolavirus antibodies that target conserved epitopes across multiple species, as well as novel antibody formats such as bispecific antibodies and nanobodies, represents a critical research frontier. The current outbreak also highlights the importance of establishing clinical trial infrastructure in outbreak settings, enabling the rapid evaluation of candidate therapeutics when they are most needed.

The citation burst history of highly influential papers further reinforces this translational narrative. As shown in **Table 5**, the most intense and sustained bursts occurred between 2012 and 2019, corresponding to the period when foundational antibody therapies were developed and clinically evaluated. For instance, the landmark ZMapp study (32) exhibited the highest burst strength, reflecting its role as a turning point in Ebola treatment research. This was preceded by early proof-of-concept studies on post-exposure antibody prophylaxis (33, 34) and followed by the pivotal randomized trial of ZMapp (35) and the PALM trial) (36)-both of which showed strong bursts extending into the early 2020s. Notably, more recent bursts (2021-2024) are associated with large-scale therapeutic trials and comprehensive reviews, while the latest burst (2024-2026) involves the FDA-approved drug Ebanga™ (37), indicating a shift from outbreak-response research to regulatory approval and clinical implementation. Collectively, this citation burst timeline aligns with the progression from basic discovery and animal model validation, through controlled human trials, to the eventual licensure of specific countermeasures. However, the absence of burst-dominating papers on pan-ebolavirus antibodies in this dataset underscores a critical gap that the current Bundibugyo outbreak has now brought into sharp focus-highlighting the urgent need for renewed research efforts targeting broadly protective therapeutics.

### 4.6 Limitations

Several limitations of this study should be acknowledged. First, bibliometric analysis is inherently descriptive and does not assess the methodological quality, clinical validity, or therapeutic efficacy of individual studies. Second, while we included three major databases, publications in regional journals, non-English languages, or emerging preprint repositories may not be fully represented, potentially introducing language and geographic bias. Third, our search strategy may have inadvertently excluded studies using alternative terminology or focusing on specific antibody therapy components without explicit mention of “antibody” or “Ebola.” Fourth, the deduplication and manual screening processes, while performed systematically, may not have captured all duplicate records or relevant articles. Fifth, the rapid evolution of Ebola research means that the most recent breakthroughs-particularly those related to the 2026 Bundibugyo outbreak-may not yet be fully reflected in the publication record. Finally, bibliometric indicators such as citation counts are influenced by field-specific citation practices and publication age and should be interpreted as proxies for impact rather than absolute measures of quality. Finally, while Google Trends data provides a novel complementary perspective on public interest in Ebola antibody therapies, this indicator has inherent limitations. It measures relative search volume from the general public rather than research activity, scientific impact, or clinical significance. Search interest is influenced by media coverage, public panic, internet accessibility, and regional variations in Google usage, and may not reflect the attention of experts, healthcare professionals, or the academic community. Therefore, our Google Trends analysis should be interpreted as a reflection of temporal patterns in public awareness, not as a proxy for scientific importance or research quality.

### 4.7 Conclusions and future directions

In conclusion, this bibliometric analysis provides a comprehensive mapping of antibody therapy research for Ebola virus disease, revealing a field characterized by outbreak-driven growth, U.S. dominance, and a clear progression from fundamental virology to clinical translation. The convergence of Ebola antibody research with broader infectious disease research, particularly COVID-19 and HIV, reflects the translational potential of antibody platform technologies and the importance of cross-disciplinary collaboration.

The 2026 Bundibugyo outbreak serves as a stark reminder that Ebola virus disease remains a significant global health threat, and that the scientific community must move beyond crisis-driven research toward sustained investment in broadly protective countermeasures. Key priorities for future research include: (1) the development of pan-ebolavirus antibody therapies effective against all species, including Bundibugyo; (2) the optimization of antibody formats, including bispecific antibodies and nanobodies, to enhance potency, breadth, and manufacturability; (3) the establishment of clinical trial infrastructure in outbreak settings to enable rapid evaluation of candidate therapeutics; (4) the exploration of combination therapies, including antibodies and antivirals, to maximize therapeutic efficacy; and (5) the translation of antibody therapies to resource-limited settings, addressing challenges of cost, cold-chain logistics, and delivery.

As the field continues to evolve, the integration of bibliometric insights with clinical and epidemiological data will be essential for guiding research prioritization, funding decisions, and public health policy. Our findings provide a systematic foundation for these efforts, offering researchers, clinicians, and policymakers a data-driven overview of the historical trajectory, current landscape, and future directions of antibody therapy for Ebola virus disease.

## Data Availability

The original contributions presented in the study are included in the article/**Supplementary Material**. Further inquiries can be directed to the corresponding authors.
